# Prediction of non emergent acute care utilization and cost among patients receiving Medicaid

**DOI:** 10.1038/s41598-023-51114-z

**Published:** 2024-01-23

**Authors:** Sadiq Y. Patel, Aaron Baum, Sanjay Basu

**Affiliations:** 1 Clinical Product Development, Waymark, San Francisco, CA USA; 2 School of Social Policy and Practice, University of Pennsylvania, 3701 Locust Walk, Philadelphia, PA 19104 USA; 3 Icahn School of Medicine at Mt Sinai, New York, NY USA; 4 Institute of Health Policy, Management and Evaluation, University of Toronto, Toronto, ON Canada; 5 Center for Vulnerable Populations, San Francisco General Hospital/University of California San Francisco, San Francisco, CA USA

**Keywords:** Health policy, Epidemiology

## Abstract

Patients receiving Medicaid often experience social risk factors for poor health and limited access to primary care, leading to high utilization of emergency departments and hospitals (acute care) for non-emergent conditions. As programs proactively outreach Medicaid patients to offer primary care, they rely on risk models historically limited by poor-quality data. Following initiatives to improve data quality and collect data on social risk, we tested alternative widely-debated strategies to improve Medicaid risk models. Among a sample of 10 million patients receiving Medicaid from 26 states and Washington DC, the best-performing model tripled the probability of prospectively identifying at-risk patients versus a standard model (sensitivity 11.3% [95% CI 10.5, 12.1%] vs 3.4% [95% CI 3.0, 4.0%]), without increasing “false positives” that reduce efficiency of outreach (specificity 99.8% [95% CI 99.6, 99.9%] vs 99.5% [95% CI 99.4, 99.7%]), and with a ~ tenfold improved coefficient of determination when predicting costs (R^2^: 0.195–0.412 among population subgroups vs 0.022–0.050). Our best-performing model also reversed the lower sensitivity of risk prediction for Black versus White patients, a bias present in the standard cost-based model. Our results demonstrate a modeling approach to substantially improve risk prediction performance and equity for patients receiving Medicaid.

## Introduction

Patients receiving Medicaid disproportionately experience social risk factors for poor health and limited access to primary care^1,2^, perpetuating health disparities between them and other populations, and resulting in high utilization of emergency departments and hospitals (‘acute care’) for non-emergent conditions^3–7^. Proactive Medicaid programs attempt to contact at-risk patients–typically based on risk models trained to predict high healthcare costs^8^—and offer patients additional support to access primary care^9^. Those programs able to contact patients deemed ‘at risk’ before they experience disease complications have successfully improved health outcomes and health equity across race/ethnic and income groups^10–13^.

Risk modeling for Medicaid has suffered from incomplete and poor quality data, lack of unification of data across states, and poor availability of metrics of social determinants of health (SDOH; such as poverty or air pollution)^14,15^. Three major questions have emerged recently from a National Academy of Medicine report related to using machine learning to improve community-based outreach to marginalized populations such as patients receiving Medicaid^16^. First, following recent multi-state efforts to improve the comprehensiveness and uniformity of data across over two dozen states, does re-training models across the newly-available unified datasets improve risk model performance? The newer data have the critical feature of linking healthcare claims (utilization and cost data) across the same individual over time with greater reliability, enabling modeling of individual healthcare trajectories, not just brief episodes of care^17^. Second, do metrics of SDOH allow us to capture complex interactions between social risk factors and healthcare utilization?^18,19^ Air pollution metrics may improve prediction of acute care for chronic lung diseases^20^, while metrics of healthy food availability may improve the prediction of acute care for diabetes^21^. While traditional logistic regression models have been used to model risk in Medicaid, newer machine learning models may better capture nonlinear and complex interactions between social determinants of health and healthcare utilization^22^. Third, can we reduce race/ethnic bias observed in models that are focused on predicting costs?^23^ Because Black patients in particular have lower access to high-cost healthcare centers such as tertiary care specialty centers, they tend to have lower costs than White patients with the same severity of disease^24^. It has been proposed that alternative modeling methods focusing on combinations of social risks and utilization rather than cost prediction alone may reduce underestimation of risk among Black patients, but the hypothesis remains untested^23^.

Here, we address these three interrelated questions for risk modeling in Medicaid. Using data from 10 million patients from states with recently-improved Medicaid data quality and comprehensiveness, we compared different modeling approaches to predict the risk of all-cause and non-emergent acute care utilization and cost.

## Methods

### Study design and conceptual model

We followed the TRIPOD guidelines for risk prediction models (Supplement Table 1). We compared: (i) conventional Medicaid risk models, which typically include patient demographic data (age, sex, and race/ethnicity), healthcare diagnostic and procedural codes, and medications as predictors (ii) models incorporating cumulative risk and risk trajectories to capture the progressive nature of chronic conditions that contribute to acute care utilization (e.g., progression of uncontrolled hypertension to heart failure); and (iii) models incorporating SDOH metrics not conventionally included in risk modeling (e.g., air pollution). Additionally, we evaluated the extent to which predictions using such metrics may be improved by newer machine learning methods that incorporate non-linearities and interaction terms, particularly as SDOH factors may interact with specific diseases to increase the risk of acute care utilization.

**Table 1:** Characteristics of patients receiving Medicaid in the study data, 2017–2019 (n = 30,619,475)*^

Characteristic group	Characteristic	N (col %)
Age category	Under 10	8,802,957 (28.7)
	10–17	4,468,984 (14.6)
	18–29	6,559,486 (21.4)
	30–39	4,237,836 (13.8)
	40–49	2,803,708 (9.2)
	50–64	3,496,766 (11.4)
	Missing	249,738 (0.8)
Sex	Female	16,306,566 (53.3)
	Male	13,917,712 (45.5)
	Missing	395,197 (1.3)
Race/ethnicity	Asian	642,057 (2.1)
	Black	5,317,920 (17.4)
	Hawaiian	236,092 (0.8)
	Hispanic	3,354,321 (11.0)
	Multiracial	55,773 (0.2)
	Native American	599,455 (2.0)
	White	12,963,558 (42.3)
	Missing	7,450,299 (24.3)
Census region^	Midwest	8,552,101 (27.9)
	Northeast	3,956,579 (12.9)
	South	10,394,744 (33.9)
	West	7,716,051 (25.2)
	Missing	0 (0.0)
Household size	Single	6,599,314 (21.6)
	2–5	8,668,539 (28.3)
	6 or more	1,148,808 (3.8)
	Missing	14,202,814 (46.4)
Federal poverty line	Under 100%	12,891,945 (42.1)
	100–200%	3,091,684 (10.1)
	200% or more	450,755 (1.5)
	Missing	14,185,091 (46.3)
English speaking	Yes	4,300,289 (14)
	No	206,961 (0.7)
	Missing	26,112,225 (85.3)
Married	Yes	1,673,105 (5.5)
	No	16,952,648 (55.4)
	Missing	11,993,722 (39.2)
US citizen	Yes	25,427,668 (83.0)
	No	1,206,701 (3.9)
	Missing	3,985,106 (13.0)
Receipt of SSI	Yes	1,801,836 (5.9)
	No	26,539,129 (86.7)
	Missing	2,278,510 (7.4)
Receipt of SSDI	Yes	705,383 (2.3)
	No	16,719,361 (54.6)
	Missing	13,194,731 (43.1)
Receipt of TANF	Yes	3,688,327 (12.0)
	No	19,579,550 (63.9)
	Missing	7,351,598 (24.0)
Disabled	Yes	1,045,831 (3.4)
	No	29,673,644 (96.6)
	Missing	0 (0.0)

*SSI* social security income, *SSDI* social security disability income, *TANF* temporary assistance for needy families.

*Data comes from Chronic Conditions Warehouse (CCW) Virtual Research Data Center (VRDC).

^We reported column percentages for all patient characteristics. All patient characteristics were provided in the TAF data. States were crosswalked to their appropriate Census Region (Supplement for distribution of patient volume by state).

### Data source

We used the Transformed Medicaid Statistical Information System Analytic Files (TAF) from 2017 to 19 (the most recent available years not affected by COVID-19), which included demographic and eligibility data, individual-level SDOH metrics, geographic information (county and zip code), and claims for outpatient, inpatient, long-term support, medication/pharmacy, and other healthcare services, including both fee-for-service and managed care.

To ensure we captured recent improvements in data comprehensiveness and quality, we included data from states meeting minimum quality standards defined by Medicaid.gov’s Data Quality Atlas^25^, which included assessment of each state’s enrollment benchmarks, claim volume, and data completeness (Supplement Section B).

### Study population, enrollment and follow-up timelines

We included individuals whose first month of enrollment in Medicaid occurred in 2017–18, to analyze their subsequent twelve months of utilization and cost, a period chosen to be directly comparable to prior Medicaid risk modeling studies^26–29^. Predictors were measured in the six month period after a patient's first month of enrollment. Outcomes were measured in the six-month period following the predictor measurement period. Also to be comparable to previous Medicaid risk modeling studies^26–29^, we excluded individuals who were dually-enrolled in both Medicare and Medicaid; Medicare covers the majority of medical services for those dually-enrolled, and dually-enrolled persons typically have separate proactive care management programs under their Medicare plans (whereas our purpose was to assist Medicaid-focused proactive outreach efforts that focus on primary care access and social services rather than elder care management; see Supplement Section C).

### Outcomes

We developed models to predict each of four outcomes: disenrollment from Medicaid (see ‘Model Comparisons’ section below), having at least one all-cause ED visit or hospitalization, having at least one non-emergent ED visit or hospitalization, and total cost of care (2019 USD per person per month, including both medical and pharmaceutical spend).

We defined ED visits based on Current Procedural Terminology codes, revenue codes, and place-of-service codes. To count “episodes” of care, we linked ED and inpatient claim records for the same patient if dates of service were congruent or contiguous^30^. We defined non-emergent ED visits as those meeting the New York University ED Patch Algorithm definition (detailed extensively in Supplement Section E)^31^. We defined non-emergent inpatient admissions as those meeting the Agency for Healthcare Research and Quality definition of a Prevention Quality Indicator (also detailed in Supplement Section E)^32^.

### Predictors

*Demographics.* We included the demographic variables available in TAF: age (in years), sex (male, female), and race/ethnicity (White, Black, Hispanic, Asian, Native American, Hawaiian, multiracial). We included race/ethnicity as a predictor because we wanted to capture the impact of systematic racism on acute care utilization; however we conducted a sensitivity analysis without race/ethnicity as a predictor to examine the impact of this choice on prediction bias between race/ethnic groups, as detailed below^33,34^. We included fixed effects for a patient’s state of residence and the year and month of their enrollment to adjust for unmeasured location, secular or seasonal factors.

*Clinical history.* From each medical or pharmaceutical claim line, we included clinical condition (principal diagnosis code), type of care (inpatient, outpatient, lab testing), clinician specialty, and medication type. Clinical conditions were defined through the Clinical Classification Software Refined categories^35^. Type of care was defined through Restructured Berenson-Eggers Type of Service System codes^36^. Clinician specialty was defined using the Centers for Medicare and Medicaid Services clinician specialty classification^37^. Medication type was defined by the CMS Prescription Drug Data Collection codes^38^.

*Cumulative risk and risk trajectories.* To capture metrics of cumulative risk and risk trajectories^39^, we included the number of: episodes for long-term non-acute care, hospitalization days, acute care visits, medication fills, and unique medications (defined as unique National Drug Codes); the percentage of acute care visits for non-emergent conditions and medication fills for generic drugs and a medication adherence measure (difference between the first and last prescription fill for a unique medication divided by total days supply). To measure risk trajectories, we included the slope for the number of all cause acute care visits, non-emergent acute care visits, and prescription fills.

*Area-level SDOH.* Using data from the 2019 AHRQ SDOH Database^40^, we included a series of area-level SDOH measures based on standard conceptual models of how SDOH factors relate to healthcare utilization^41^. We included measures of social conditions, health care resources, environmental factors, and per capita rates of death (list in Supplement Section G). TAF provided both zip and county codes, but we conducted analyses at the county level due to extensive literature showing limited added explanatory power of zip code SDOH measures for predicting health outcomes for patients on Medicaid, and because Medicaid outreach programs are often organized at the county government level^42,43^.

*Individual-level SDOH.* We included the individual-level SDOH variables available in TAF: house size (single, 2–5, 6 or more), income level (0–100% federal poverty level, 100–200%, and 200% or more), and binary indicators (yes/no) for English speaking, married, US citizen, recipient of supplemental security income, recipient of social security disability insurance, recipient of Temporary Assistance for Needy Families, and whether the person gained Medicaid eligibility due to disability. Due to variation in missing data for patient characteristics (detailed in the Supplement Section H), we included a missing category for each characteristic instead of imputing missing data, per recent guidelines concerning informative missingness. In particular, this approach acknowledges that the presence of missingness itself may provide valuable information for predicting acute care utilization (e.g., persons refusing to answer a US Citizen question may be disproportionately unwilling to register in government-sponsored community health centers, affecting primary care utilization)^44^.

We transformed all continuous variables in our model with a standard scaler and all categorical variables with one-hot encoding^45^.

### Model comparisons

Because of high disenrollment rates in Medicaid^46^, we conducted a two-stage model (see Supplement Section K for details). We randomly split our sample into two parts. In the first stage, using the first part of our sample, we modeled each patient’s probability of disenrollment (see Supplement Section L for model specification)^47^. In the second stage, using the second part of our sample, for each member, we first predicted the probability of their disenrollment using the model weights of the top-performing model in the first stage. Second, we modeled the non-emergent acute care utilization probability, conditional on their predicted disenrollment and other covariates (see Supplement Section L for model specification). Rather than narrowly restricting analysis only to people with long term continuous coverage, this two-stage procedure permits greater generalizability by explicitly modeling the risk of coverage loss and capturing interactions between other covariates and which patients come in and out of coverage (n.b., 25% of study participants lost coverage within 12 months, with wide variation across states; Supplement Tables 2 and 3).

**Table 2:** Comparative effectiveness of models predicting utilization of non-emergent acute care visits*^

Model and outcome	AUC (95% CI)	Accuracy (95% CI)	MCC (95% CI)	F1 Score (95% CI)	Sensitivity (95% CI)	Specificity (95% CI)	NPV (95% CI)	PPV (95% CI)
**Logistic regression**								
Baseline comparison model	0.699 (0.690, 0.709)	0.893 (0.891, 0.896)	0.105 (0.090, 0.123)	0.065 (0.056, 0.074)	0.034 (0.030, 0.040)	0.995 (0.994, 0.997)	0.896 (0.895, 0.900)	0.476 (0.432, 0.532)
Cumulative risk and trajectories measures	0.754 (0.748, 0.761)	0.896 (0.893, 0.898)	0.171 (0.156, 0.187)	0.122 (0.110, 0.133)	0.067 (0.061, 0.074)	0.994 (0.993, 0.995)	0.900 (0.897, 0.901)	0.575 (0.535, 0.615)
Area SDOH	0.755 (0.748, 0.762)	0.895 (0.893, 0.895)	0.172 (0.156, 0.186)	0.122 (0.110, 0.134)	0.068 (0.061, 0.077)	0.994 (0.993, 0.995)	0.899 (0.897, 0.900)	0.575 (0.533, 0.611)
Area and individual SDOH	0.757 (0.750, 0.762)	0.895 (0.892, 0.898)	0.169 (0.154, 0.184)	0.120 (0.108, 0.131)	0.067 (0.064, 0.070)	0.994 (0.993, 0.995)	0.899 (0.897, 0.902)	0.568 (0.532, 0.608)
**Regularized logistic regression**								
Baseline comparison model	0.698 (0.691, 0.706)	0.894 (0.892, 0.896)	0.105 (0.089, 0.118)	0.063 (0.054, 0.071)	0.034 (0.029, 0.038)	0.996 (0.995, 0.997)	0.897 (0.894, 0.899)	0.477 (0.423, 0.521)
Cumulative risk and trajectories measures	0.754 (0.747, 0.761)	0.896 (0.893, 0.898)	0.172 (0.158, 0.188)	0.122 (0.111, 0.134)	0.068 (0.062, 0.076)	0.994 (0.993, 0.995)	0.900 (0.897, 0.902)	0.576 (0.540, 0.618)
Area SDOH	0.754 (0.748, 0.761)	0.896 (0.893, 0.897)	0.169 (0.154, 0.186)	0.122 (0.111, 0.133)	0.067 (0.062, 0.074)	0.994 (0.993, 0.995)	0.900 (0.897, 0.902)	0.569 (0.533, 0.610)
Area and individual SDOH	0.755 (0.749, 0.762)	0.896 (0.893, 0.899)	0.173 (0.157, 0.188)	0.122 (0.111, 0.132)	0.069 (0.062, 0.075)	0.994 (0.993, 0.995)	0.900 (0.897, 0.903)	0.576 (0.538, 0.613)
**Random forests**								
Baseline comparison model	0.710 (0.703, 0.718)	0.895 (0.892, 0.897)	0.101 (0.085, 0.112)	0.024 (0.018, 0.029)	0.012 (0.009, 0.015)	0.999 (0.998, 1.000)	0.894 (0.892, 0.897)	0.945 (0.871, 0.986)
Cumulative risk and trajectories measures	0.750 (0.740, 0.754)	0.897 (0.894, 0.899)	0.169 (0.140, 0.170)	0.061 (0.058, 0.076)	0.035 (0.027, 0.037)	0.999 (0.998, 1.000)	0.897 (0.894, 0.900)	0.920 (0.830, 0.924)
Area SDOH	0.754 (0.747, 0.762)	0.897 (0.894, 0.900)	0.166 (0.150, 0.177)	0.067 (0.058, 0.076)	0.036 (0.030, 0.040)	0.999 (0.998, 1.000)	0.897 (0.894, 0.900)	0.881 (0.851, 0.937)
Area and individual SDOH	0.759 (0.752, 0.767)	0.898 (0.895, 0.900)	0.175 (0.159, 0.186)	0.071 (0.061, 0.081)	0.038 (0.032, 0.042)	0.999 (0.998, 1.000)	0.897 (0.895, 0.900)	0.924 (0.880, 0.953)
**XGBoost**								
Baseline comparison model	0.717 (0.711, 0.727)	0.897 (0.895, 0.900)	0.171 (0.156, 0.184)	0.070 (0.061, 0.079)	0.036 (0.032, 0.042)	0.999 (0.998, 1.000)	0.897 (0.895, 0.900)	0.911 (0.872, 0.947)
Cumulative risk and trajectories measures	0.783 (0.777, 0.788)	0.901 (0.898, 0.903)	0.234 (0.220, 0.254)	0.154 (0.142, 0.167)	0.083 (0.080, 0.089)	0.997 (0.996, 0.998)	0.902 (0.899, 0.904)	0.779 (0.748, 0.813)
Area SDOH	0.793 (0.790, 0.795)	0.903 (0.899, 0.904)	0.287 (0.273, 0.300)	0.155 (0.143, 0.168)	0.107 (0.103, 0.116)	0.998 (0.997, 0.999)	0.903 (0.900, 0.905)	0.810 (0.780, 0.841)
Area and individual SDOH	0.795 (0.781, 0.795)	0.904 (0.902, 0.907)	0.293 (0.279, 0.306)	0.164 (0.152, 0.178)	0.113 (0.105, 0.121)	0.998 (0.996, 0.999)	0.905 (0.902, 0.908)	0.811 (0.775, 0.836)

*AUC* area under the ROC curve, *MCC* Matthews correlation coefficient, *NPV* negative predictive value, *PPV* positive predictive value, *XGBoost* extreme gradient boosting.

*We sequentially compared multiple alternative models to compare the benefits of including different predictor variables and the impact of alternative model fitting algorithms. First, we devised a model with demographics and clinical history to mimic standard risk models (named ‘baseline comparison model’). Second, we developed a model with cumulative risk and risk trajectories variables (named the ‘cumulative risk and trajectories’ model). Finally, we created two additional models (including all measures in the ‘cumulative risk and trajectories’ model), one with area-level SDOH predictors (named, ‘area SDOH’) and another with both area- and individual-level SDOH predictors (named ‘area and individual SDOH’). See Supplement Section L for model specification.

^**^**^Predictors were measured in the six-month period after a patient’s first month of enrollment in Medicaid from 2017 to 18. The outcome was a binary (yes/no) indicator of whether a patient had at least 1 non-emergent acute care visit in the 6-month period immediately following the 6-month predictor measurement period. See Supplement Section M for machine learning modeling.

**Table 3:** Comparative effectiveness of models predicting utilization of all-cause acute care visits.

Model and outcome	AUC (95% CI)	Accuracy (95% CI)	MCC (95% CI)	F1 Score (95% CI)	Sensitivity (95% CI)	Specificity (95% CI)	NPV (95% CI)	PPV (95% CI)
**Logistic regression**								
Baseline comparison model	0.700 (0.693, 0.706)	0.800 (0.797, 0.804)	0.211 (0.202, 0.224)	0.212 (0.202, 0.221)	0.127 (0.121, 0.134)	0.979 (0.978, 0.980)	0.808 (0.805, 0.812)	0.612 (0.597, 0.637)
Cumulative risk and trajectories measures	0.755 (0.749, 0.760)	0.811 (0.808, 0.814)	0.294 (0.283, 0.305)	0.315 (0.304, 0.325)	0.206 (0.198, 0.214)	0.972 (0.971, 0.974)	0.821 (0.818, 0.825)	0.663 (0.646, 0.678)
Area SDOH	0.756 (0.751, 0.761)	0.812 (0.808, 0.814)	0.295 (0.283, 0.304)	0.315 (0.305, 0.236)	0.207 (0.199, 0.215)	0.972 (0.971, 0.973)	0.822 (0.818, 0.825)	0.662 (0.645, 0.677)
Area and individual SDOH	0.757 (0.752, 0.762)	0.812 (0.809, 0.815)	0.295 (0.284, 0.306)	0.317 (0.307, 0.327)	0.209 (0.201, 0.217)	0.971 (0.970, 0.973)	0.822 (0.820, 0.826)	0.660 (0.644, 0.676)
**Regularized logistic regression**								
Baseline comparison model	0.700 (0.695, 0.707)	0.800 (0.797, 0.803)	0.213 (0.202, 0.224)	0.212 (0.203, 0.221)	0.128 (0.122,0.135)	0.979 (0.977, 0.980)	0.808 (0.805, 0.812)	0.616 (0.595, 0.634)
Cumulative risk and trajectories measures	0.756 (0.750, 0.761)	0.811 (0.808 ,0.815)	0.295 (0.283, 0.305)	0.315 (0.304, 0.324)	0.207 (0.199, 0.214)	0.972 (0.970, 0.974)	0.822 (0.819, 0.825)	0.662 (0.646, 0.679)
Area SDOH	0.755 (0.751, 0.761)	0.811 (0.807, 0.814)	0.294 (0.284, 0.304)	0.315 (0.305, 0.325)	0.207 (0.200, 0.215)	0.972 (0.970, 0.973)	0.821 (0.818, 0.824)	0.663 (0.645, 0.677)
Area and individual SDOH	0.756 (0.751, 0.762)	0.811 (0.808, 0.814)	0.293 (0.283, 0.305)	0.316 (0.306, 0.325)	0.207 (0.200, 0.216)	0.971 (0.970, 0.973)	0.822 (0.818, 0.825)	0.659 (0.644, 0.676)
**Random forests**								
Baseline comparison model	0.711 (0.706, 0.717)	0.803 (0.800, 0.807)	0.223 (0.216, 0.236)	0.141 (0.134, 0.152)	0.077 (0.073, 0.083)	0.997 (0.996, 0.998)	0.802 (0.798, 0.806)	0.874 (0.861, 0.903)
Cumulative risk and trajectories measures	0.746 (0.745, 0.758)	0.817 (0.815, 0.822)	0.313 (0.312, 0.333)	0.273 (0.269, 0.289)	0.164 (0.160, 0.174)	0.990 (0.989, 0.993)	0.817 (0.814, 0.820)	0.820 (0.819, 0.858)
Area SDOH	0.757 (0.750, 0.761)	0.820 (0.816, 0.822)	0.331 (0.317, 0.337)	0.286 (0.274, 0.295)	0.172 (0.165, 0.179)	0.992 (0.990, 0.993)	0.819 (0.815, 0.821)	0.853 (0.824, 0.854)
Area and individual SDOH	0.756 (0.753, 0.760)	0.814 (0.813, 0.821)	0.331 (0.326, 0.335)	0.275 (0.272, 0.295)	0.166 (0.164, 0.177)	0.986 (0.984, 0.993)	0.817 (0.816, 0.819)	0.852 (0.846, 0.858)
**XGBoost**								
Baseline comparison model	0.717 (0.711, 0.723)	0.821 (0.817, 0.824)	0.338 (0.328, 0.348)	0.295 (0.286, 0.306)	0.179 (0.172, 0.187)	0.992 (0.991, 0.993)	0.819 (0.816, 0.823)	0.849 (0.835, 0.863)
Cumulative risk and trajectories measures	0.776 (0.770, 0.781)	0.822 (0.818, 0.824)	0.349 (0.339, 0.359)	0.368 (0.358, 0.378)	0.247 (0.239, 0.255)	0.974 (0.973, 0.975)	0.830 (0.826, 0.833)	0.720 (0.706, 0.734)
Area SDOH	0.807 (0.800, 0.809)	0.838 (0.835, 0.841)	0.419 (0.418, 0.437)	0.432 (0.422, 0.442)	0.287 (0.285, 0.302)	0.982 (0.981, 0.984)	0.841 (0.836, 0.843)	0.810 (0.807, 0.834)
Area and individual SDOH	0.805 (0.798, 0.806)	0.834 (0.831, 0.837)	0.415 (0.411, 0.429)	0.429 (0.419, .439)	0.288 (0.270, 0.286)	0.981 (0.979, 0.982)	0.837 (0.832, 0.839)	0.807 (0.804, 0.829)

*AUC* area under the ROC curve, *MCC* Matthews correlation coefficient, *NPV* negative predictive value, *PPV* positive predictive value, *XGBoost* extreme gradient boosting.

*We sequentially compared multiple alternative models to compare the benefits of including different predictor variables and the impact of alternative model fitting algorithms. First, we devised a model with demographics and clinical history to mimic standard risk models (named ‘baseline comparison model’). Second, we developed a model with cumulative risk and risk trajectories variables (named the ‘cumulative risk and trajectories’ model). Finally, we created two additional models (including all measures in the ‘cumulative risk and trajectories’ model), one with area-level SDOH predictors (named, ‘area SDOH’) and another with both area- and individual-level SDOH predictors (named ‘area and individual SDOH’). See Supplement Section L for model specification.

^^^Predictors were measured in the six-month period after a patient’s first month of enrollment in Medicaid from 2017 to 18. The outcome was a binary (yes/no) indicator of whether a patient had at least 1 all-cause acute care visit in the 6-month period immediately following the 6-month predictor measurement period. See Supplement Section M for machine learning modeling.

For the second stage, we compared multiple predictor variable combinations to assess the value of collecting and integrating different types of predictors into Medicaid risk models. First, we developed a baseline model with demographics and clinical history (referred to as the ‘Baseline comparison model’). Second, we created a model incorporating cumulative risk and risk trajectories (the ‘Cumulative risk and trajectories’ model). Third, we built two additional models, one including area-level SDOH predictors (‘Area SDOH’) and another incorporating both area- and individual-level SDOH predictors (‘Area and individual SDOH’) to evaluate the added value of collecting individual-level SDOH measures. Each stage 2 model (i.e., ‘Baseline comparison’ to ‘Area and individual SDOH’) included the patient’s probability of disenrollment.

### Model fitting algorithms

We applied four model fitting algorithms to both the first and second stage of analysis, based on our conceptual model and key debates in the Medicaid research landscape concerning risk modeling: standard regressions, regularized regressions with elastic net regularization, random forest (RF), and extreme gradient boosting (XGBoost). We selected standard regressions–logistic regression for the binary acute care utilization outcome measure and linear regression for the transformed cost outcome measure–as both are the modeling approaches used by common Medicaid risk models^8^. Given the large number of predictors in our risk model compared to conventional risk models, we used a regularized regression model, using elastic-net regularization (which combines the benefits of ridge and LASSO regression) for feature selection and to minimize the effect of outliers among collinear variables^48^. Next, we selected RF, a large-scale averaging or ‘bagging’ learning algorithm. Finally, we selected extreme gradient boosting, or XGBoost, as a machine learning algorithm to compare to standard and regularized logistic regression as well as RF^49–52^. We implemented the targeted hyperparameter tuning method proposed by Van Rijn and Hutter for feature selection in XGBoost and RF to improve performance in tuning (Supplement Section M)^53^.

We implemented the following strategies to accommodate CMS computing and runtime rules. First, we used PySpark, as it processes big data in less time compared to Python. Second, the specific PySpark module available in the CMS data center inefficiently executed k-fold validation; thus, we used a simple hold-out validation. Finally, we took a random sample of 10 million of the 30.6 million patients. Because we had a population sample for each state to help capture state variations known to be important in Medicaid, we believed bias would be minimized. The distribution of predictors and outcomes for both samples was identical, as measured by their standardized mean differences of < 0.01 (Supplement Section N).

### Comparison to cost-based risk model

To predict cost among patients in the six states that provided cost data, we compared our model’s performance to the widely used Chronic Illness and Disability Payment System (CDPS, version 7.0)^54,55^. We used the same modeling approach for predicting cost as described above for predicting acute care utilization. CPDS predicted cost using a linear regression model with predictors of patient demographics (age, sex, race/ethnicity), diagnostic codes, and medications^54^.

### Performance measures

We calculated the Matthews Correlation Coefficient (MCC, a metric combining the sensitivity [true positive proportion] and specificity [true negative proportion]) as the overall measure for model performance, as it is less sensitive than C-statistic to minor model improvements^56^. We additionally reported the F1 score, a composite of a model’s precision and recall scores, and C-statistic (or area under the curve, AUC), a ‘discrimination’ metric indicating how well the models predicted higher-risk patients, and model accuracy, or the proportion of all predictions that were correct. For completeness, we included two additional metrics commonly used by clinical epidemiologists: the positive predictive value (PPV), or proportion of those flagged ‘at risk’ who truly subsequently experienced the outcome in the follow-up period, and negative predictive value (NPV), or proportion flagged ‘not at risk’ who truly did not subsequently experience the outcome. 95% confidence intervals were estimated around each metric via bootstrap (Supplement Section M)^57^.

### Bias and sensitivity

We assessed ethnic/racial bias using the equalized odds method, which quantifies inequalities in sensitivity and specificity across groups for prediction of all-cause and non-emergent acute care visits^58^. We also compared predicted and observed costs per member per month by race/ethnicity to evaluate bias in cost prediction.

We repeated our analysis after removing race/ethnicity from our predictor variables to test the hypothesis that underestimation of risk for minorities may increase after race/ethnicity is eliminated from the model, because other variables can underpredict risk for minorities due to inadequate capture of the effect of systemic racism on healthcare utilization patterns^23^. Further, because 40 percent of our sample consisted of White patients, we also separately evaluated the impact of downsampling White patients (effectively upsampling minority patients relative to White patients) to reduce race/ethnic prediction bias (Supplement).

Given the large volume of children in our sample, we also repeated our analysis with adults only, as we recognize that most of the non-emergent ED visits and hospitalizations in our study would be among adults (Supplement Section O).

Because class imbalanced data hinders classification performance of RF^49^, for the best performing RF model, we performed a downsampling procedure, specifically training on a disproportionately lower subset of patients with no acute care events (Supplement).

This study was approved by the Western Institutional Review Board. All methods were performed in accordance with the relevant guidelines and regulations. Informed consent for this study was waived by the Western Institutional Review Board. The datasets utilized in this study are not publicly accessible. However, they can be obtained from the Centers for Medicare and Medicaid Services. Accessing this data entails a comprehensive procedure, involving completion of an Institutional Review Board (IRB) process and the procurement of a seat on their data portal. Researchers who possess a seat on the data portal can obtain the code necessary to replicate our study findings from the Github repository listed at the end of this manuscript. Model construction and comparison were performed in PySpark (version 3.2.1).

## Results

Data from 26 states and Washington DC, with a total of 30,619,475 unique patients, met comprehensiveness and quality metrics for inclusion in the study (Table 1). The majority of patients identified as female (53.3%), were under 18 years of age (64.7%), US citizens (83.0%), not married (55.4%), and did not indicate having a disability (96.6%). Under half were White (42.3%), and under half were living below the federal poverty line (42.1%). In the 12-month period following enrollment, 24.6% of patients lost Medicaid coverage; 21.0% of patients had at least one all-cause acute care visit, and 10.6% of all patients (50.5% of those patients with at least one all-cause acute care visit) had at least one non-emergent acute care visit (Supplement Tables 2 and 3). Key covariate distributions did not differ between the full sample and the 10 million person random subsample that we used for modeling (Supplement Section N).

In our first stage analysis, XGBoost was the best performing model for predicting loss of Medicaid coverage (see Supplement Tables 4 and 5 for comprehensive metrics across all stage-one models). The standard comparison model (logistic regression) had an AUC/C-statistic of 69.4% (95% CI 69.0, 70.1%) versus regularized regression of 69.5% (95% CI 69.0, 70.1%), RF of 73.9% (95% CI 73.8, 74.3%), and XGBoost of 74.9% (95% CI 74.4, 75.3%); the baseline model had a sensitivity of 11.4% (95% CI 11.0, 12.1%) versus regularized regression of 11.5% (95% CI 10.8, 12.0%), RF of 17.0% (95% CI 16.9, 17.5%), and XGBoost of 20.9% (95% CI 20.3, 21.7%); the baseline model had a specificity of 98.3% (95% CI 98.2, 98.4%) versus regularized regression of 98.3% (95% CI 98.2, 98.4%), RF of 98.3% (95% CI 98.2, 98.4%), and XGBoost of 97.5% (95% CI 97.3, 97.6%); and the baseline model had a MCC of 21.1% (95% CI 20.3, 22.4%) versus regularized regression of 21.2% (95% CI 20.0, 22.1%), RF of 29.3% (95% CI 28.9, 29.7%), and XGBoost of 30.9% (95% CI 30.0, 31.9%).

### Baseline comparison model for predicting acute care utilization

In our second stage analysis, for predicting non-emergent acute care visits, our baseline comparison model included demographics and clinical history. For predicting non-emergent acute care visits, standard logistic regression had similar results to regularized logistic regression, while RF had lower sensitivity and MCC (performing worst among the models). XGBoost had a higher performance than standard logistic regression for discrimination (C-statistic, 71.7; 95% CI 71.1–72.7; 1.8 percentage point increase from using XGBoost versus standard logistic regression; 95% CI 1.8–2.1); sensitivity (3.6%, 95% CI 3.2–3.4%; 0.2 percentage point increase; 95% CI 0.2–0.2); specificity (99.9; 95% CI 99.8–100.0; 0.4 percentage point increase; 95% CI 0.3–0.4); and MCC (17.1; 95% CI 15.6–18.4; 6.6 percentage point increase; 95% CI 6.1–6.6; Table 2 and Supplement Fig. 1). Parallel results were observed when predicting all-cause acute care visits (Table 3 and Supplement Fig. 2).

### Improvement from including cumulative risk and trajectories predictors

Adding cumulative risk and risk trajectories to the models improved their discrimination, sensitivity and MCC without reducing specificity. For predicting non-emergent acute care, including cumulative risk and risk trajectory predictors in the highest-performing model (XGBoost) resulted in a gain in discriminative ability (C-statistic, 6.6 percentage point increase; 95% CI 6.1–6.6); sensitivity (4.7 percentage point increase; 95% CI 4.6–4.9); and MCC (6.7 percentage point increase; 95% CI 6.7–7.0; Table 2 and Supplement Fig. 1). There was a small decrease in specificity (0.2 percentage point decrease; 95% CI 0.2–0.2). Parallel results were observed when predicting all-cause acute care visits (Table 3 and Supplement Fig. 2).

### Improvement from including area- and individual-level SDOH predictors

There was no net improvement after including area or individual SDOH predictors for both logistic, regularized logistic regression or RF models, but there was a significant improvement for XGBoost models. XGBoost produced a net improvement after including area SDOH predictors for discriminative ability (C-statistic, 1.0 percentage point increase; 95% CI 0.7–1.3); sensitivity (2.4 percentage point increase; 95% CI 2.3–2.7); and MCC (5.3 percentage point increase; 95% CI 4.6–5.3; Table 2 and Supplement Fig. 1). There was no significant change for specificity. Additionally, there was no further significant change after including individual SDOH predictors beyond including area-level SDOH predictors. Parallel results were observed when predicting all-cause acute care visits (Table 3 and Supplement Fig. 2).

### Improvement from using XGBoost

Focusing on the best performing model by MCC overall–the model with all clinical predictors, cumulative risk and risk trajectories measures, and area-level SDOH indicators–we measured the net improvement from using XGBoost compared to logistic regression (standard or regularized), as logistic regression performed better than RF and is the current standard modeling approach. For predicting non-emergent acute care visits (Table 2 and Supplement Fig. 1), XGBoost had a net improvement versus logistic regression for discriminative ability (C-statistic, 3.8 percentage point increase over standard; 95% CI 3.3–4.2; 3.9 percentage point over regularized; 95% CI 3.4–4.2); sensitivity (3.9 percentage point increase over standard; 95% CI 3.9–4.2; 4.0 percentage point over regularized; 95% CI 4.0–4.1); specificity (0.4 percentage point increase over standard; 95% CI 0.4–0.4; 0.4 percentage point over regularized; 95% CI 0.4–0.4); and MCC (11.5 percentage point increase over standard; 95% CI 11.4–11.7; 11.8 percentage point over regularized; 95% CI 11.4–11.9). Parallel results were observed when predicting all-cause acute care visits (Table 3 and Supplement Fig. 2).

### Performance of the best performing model

The best performing model by MCC overall was XGboost with cumulative risk and risk trajectory measures and area-level SDOH measures. The model had an overall performance for predicting non-emergent acute care visits (Supplemental Table 6) and tripled the probability of prospectively identifying at-risk patients versus the standard logistic regression without risk trajectory or SDOH measures (sensitivity 11.3% [95% CI 10.5, 12.1%] vs 3.4% [95% CI 3.0, 4.0%]), without increasing “false positives” (specificity 99.8% [95% CI 99.6, 99.9%] vs 99.5% [95% CI 99.4, 99.7%]).

### Variable importance

Variables of highest importance for the best-performing model by MCC (XGBoost) were estimated by the Gini index, are shown in Supplement Fig. 3. Complex medical disorders (e.g., sequelae of cerebral infarction), having a higher probability of losing Medicaid, participating in behavioral health services, and several SDOH variables (e.g., poor air quality days) were key variables for predicting acute-care visits. Poor air quality and respiratory conditions commonly interacted, as did behavioral and specific somatic conditions such as cardiac and gastrointestinal conditions (Supplement Tables 8 and 9).

### Comparison of cost-based models

The six states reporting cost data had a total sample size of 2,627,775 unique individuals. In this sample, the CDPS R^2^ statistic varied from 0.022 to 0.050 across adults, children, and people with disabilities, while the best performing model (XGBoost with cumulative risk and risk trajectories and area-level SDOH metrics) outperformed CDPS in terms of the coefficient of determination by roughly tenfold (R^2^ statistic ranged 0.265–0.412 across the different population groups; Supplement Table 9).

CDPS underpredicted cost per member per month for Black patients and overestimated for White patients, with differences ranging from $11–$46 (*p* < 0.001), whereas the best performing XGBoost cost-predicting model narrowed these differences ($5–$25, *p* < 0.001; Supplement Table 10). Results for Hispanic and other minority groups were inconsistent, with a range of over- and under-prediction across subgroups for all models (Supplement Table 10).

### Bias and sensitivity

For the best performing model of non-emergent acute care utilization by MCC, there was higher sensitivity for Black patients than White patients, but lower sensitivity for Hispanic and other minority patients than White patients (White: 0.089; 95% CI 0.088–0.090; Black: 0.097; 95% CI 0.096–0.099; Hispanic: 0.065; 95% CI 0.064–0.068; other: 0.063; 95% CI 0.059–0.066). There were minimal differences in specificity by race/ethnicity (Supplement Table 10).

When removing race/ethnicity as a predictor variable, the model sensitivity reduced for Black patients, although there was still overall a higher sensitivity for Black patients compared to White patients when modeling utilization and incorporating our XGBoost modeling approach incorporating risk trajectories and SDOH variables (Supplement Table 11), with no effect on specificity. After downsampling White patients, similar patterns persisted although smaller in magnitude; however, the White-Hispanic and White-other minority group difference in sensitivity reduced (Supplement Table 12).

When removing children from the dataset to focus only on adults, we observed similar performance for predicting non-emergent acute care visits as when including both children and adults (Supplement Table 13).

After performing a downsampling procedure for best performing RF model, the model MCC, sensitivity, and F1-score increased, enabling RF to outperform logistic regression, but not XGBoost; however, there was decreased specificity of the RF model (Supplement Table 14).

## Discussion

In applying a series of newer modeling techniques to a 10 million person sample of Medicaid patients across multiple states that have made substantial efforts to improve their data comprehensiveness and quality, we achieved the largest and most generalizable Medicaid risk model comparison to date (as the previously largest analysis was limited to N = 3.9 million people, with no accounting of race/ethnicity, across seven states, versus our analysis of 10 million people with 42% non-White across 26 states and Washington DC that is helpful given state-specific variations in Medicaid administration)^26,59^, and achieved higher performance than any other Medicaid risk model in the field (with our best-performing model having an AUC/C-statistic of 79.5% for non-emergent acute care [95% CI 78.1, 79.5%], versus the 67.7% highest AUC/C-statistic reported in the literature [no 95% CI reported, and the other metrics we reported here were also not previously reported])^60^. For predicting non-emergent acute visits, the best-performing model tripled the probability of prospectively identifying at-risk patients versus a standard model, without increasing “false positives” that could reduce the efficiency of Medicaid outreach programs limited by time, funding and personnel. When predicting costs, our best-performing model also outperformed the most common model used by Medicaid to date (CDPS) by ~ tenfold in terms of the coefficient of determination.

Incorporating cumulative risk and risk trajectories based on improvements to Medicaid data substantially improved model performance, as did the incorporation of SDOH metrics–although the latter only improved models that used a specific type of machine learning model to capture complex nonlinearities and interaction terms not included in standard logistic regressions currently used by Medicaid state agencies and health plans. Contrary to our expectations, inclusion of individual-level SDOH metrics did not further improve performance of our models beyond area-level SDOH metrics–potentially due to missingness in TAF datasets of key individual-level SDOH metrics most associated with acute care utilization, such as food and housing insecurity^61^. These findings can inform ongoing efforts to collect more relevant SDOH data. Importantly, our XGBoost machine learning model also captured complex interactions of behavioral health and somatic health conditions, which are known in the literature to increase non-emergent acute care^62^, but are not currently included in common Medicaid risk prediction models.

We found that our modeling approach reversed the lower sensitivity of risk prediction for Black versus White patients, a bias present in the standard cost-based model, though it did not fully resolve other minority-White prediction biases. This finding persisted even after removing race/ethnicity as a predictor variable, suggesting that other predictors in the model (e.g., SDOH variables) and the modeling approach itself addressed bias in predicting risk for Black patients. One persistent challenge in developing risk models is that claims data typically reflect higher healthcare access among White patients^63^. Our modeling approach is one strategy to mitigate this challenge, offering a possible approach to more equitable application of machine learning to Medicaid risk modeling^64^.

Our analysis has several limitations. First, we excluded 23 states with insufficient data comprehensiveness or quality, though our study is more inclusive compared to previous studies^9^. Second, we utilized claims-based algorithms to categorize acute care visits as non-emergent, which may overlook contextual factors that influence such utilization^65^. Third, we used data from 2017 to 2019 instead of 2020 due to COVID-19. Recalibration to address utilization pattern variations due to COVID-19 may be useful whenever newer data are released. Fourth, our model excluded dually-eligible Medicare and Medicaid patients, as their claims are primarily in Medicare data and they typically have Medicare-oriented outreach programs without Medicaid-specific components (e.g., pediatrics, maternity).

In the future, as more researchers utilize the newly-available Medicaid data, a collaborative federated learning network may facilitate improved model sharing and comparisons for Medicaid. Future research may also focus on developing and validating cohort-specific (e.g., maternity, pediatric) models and state-specific models to compare group and geography-specific modeling performance.

Our current findings nevertheless demonstrate the opportunity to improve models to support proactive outreach programs for patients receiving Medicaid, for whom data and services have traditionally lagged behind Medicare and commercial insurance markets and whose differential access to quality care has perpetuated health disparities across race/ethnic and income groups across the United States.

### Supplementary Information

Supplementary Information.

## Data Availability

The datasets utilized in this study are not publicly accessible. However, they can be obtained from the United States federal agency, the Centers of Medicare and Medicaid. Accessing this data entails a comprehensive procedure, involving completion of an Institutional Review Board (IRB) process and the procurement of a seat on their data portal. Researchers can find code necessary to replicate and extend our study findings on GitHub: https://github.com/sadiqypatel/.Medicaid_Risk_Model.
