# Supplementary material for: Prediction of non emergent acute care utilization and cost among patients receiving Medicaid
Source: Sci Rep. 2024 Jan 23;14:824. doi: 10.1038/s41598-023-51114-z (PMC10805799; doi:10.1038/s41598-023-51114-z)
Supplement: Supplementary file 1 — Supplementary Information. [file 41598_2023_51114_MOESM1_ESM.docx]

**Table of Contents**

**Methods**

1. **Medicaid Data**
2. **Data quality assessment**
   1. Overview
   2. Part 1: Enrollment benchmarks and claims volume
   3. Part 2: Utilization measures
   4. Part 3: Spending measures
3. **Exclusion Criteria**
4. **Distribution of final sample by state**
5. **Defining acute care visits**
   1. Identifying acute care visits
   2. Defining Non-emergent ED Visits
   3. Defining Non-emergent Hospitalizations
   4. Defining Episodes of Care
6. **Defining clinician specialty**
7. **Area-level Measures of Social Determinants of Health**
8. **Person-level Measures of Social Determinants of Health**
9. **Defining members with a disability**
10. **Prediction and Outcome Measurement Periods**
11. **Defining two-stage procedure**
    1. Overview
    2. Stage 1: Predicting likelihood of losing Medicaid coverage
    3. Stage 2: Predicting likelihood of acute care visit
12. **Model Specification**
    1. Stage 1 Model
    2. Stage 2 Model
13. **Machine learning procedure**
    1. Overview
    2. Train, validate, and test samples
    3. Point Estimates and 95% Confidence Intervals
    4. Hyperparameters
14. **Distribution of predictors and outcomes for total sample of versus random sample of patients**
15. **Sensitivity Analyses**

**Appendix Tables**

1. **Table 1:** Transparent reporting of a multivariable prediction model for individual prognosis or diagnosis (TRIPOD): The TRIPOD statement
2. **Table 2:** Total number of months enrolled in Medicaid in 12-month period after first month of enrollment from 2017-18
3. **Table 3:** Percentage of patients who lose Medicaid Coverage within a 12-month period by State
4. **Table 4:** Model Performance for Stage 1 analysis [predicting loss of Medicaid coverage]
5. **Table 5:** Importance scores for predicting loss of Medicaid coverage
6. **Table 6:** Correlations among top 15 most important predictors for non-emergent acute care visits
7. **Table 7:** Correlations among top 15 most important predictors for all-cause acute care visits
8. **Table 8:** Models predicting patient cost
9. **Table 9:** Assessment of racial bias for predicting cost
10. **Table 10:** Assessment of racial bias for predicting acute care utilization
11. **Table 11:** Sensitivity analysis for assessment of racial bias (removing race from model)
12. **Table 12:** Sensitivity analysis for assessment of racial bias (downsampling White patients)
13. **Table 13** Sensitivity analysis for adults only
14. **Table 14** Sensitivity analysis for adults only
15. **Figure 1:** Comparative effectiveness of models predicting utilization of all-cause acute care visits
16. **Figure 2:** Comparative effectiveness of models predicting utilization of non-emergent acute care visits
17. **Figure 3:** Importance of top 15 predictors in the best performing model (XGBoost model with clinical, cumulative risk and trajectories, and area-level SDOH measures)

**Methods**

1. **Medicaid data**

The Medicaid and CHIP TMSIS Analytic Files (TAF) provides the 100% claims sample for all Medicaid members. It includes fee-for-service and managed care Medicaid members. *Our study used 2017-2019 data from the following files:*

- *Demographic and enrollment*
  - Contains demographic, eligibility, and enrollment characteristics of beneficiaries who were enrolled in Medicaid or in CHIP for at least one day during any given calendar year
- *Inpatient*
  - Contains records for enrollees who had an inpatient hospital stay
- *Other services*
  - Includes physician services, outpatient hospital institutional utilization, lab/X-ray, clinic services, home health, hospice and premium payments
- *Pharmacy*
  - Includes records of filled prescriptions
- *Long-term care*
  - Contains records for institutional long term care provided by specific facilities identified by the Type of Service variable: Nursing Facility Services. Mental Facility Services

1. **Data quality assessment**

**Overview**



**Part 1: Enrollment benchmarks and claims volume**

First, for 2017-2019, we eliminated states that did not meet the DQ Atlas^1^ low or medium concern standards for enrollment benchmarks and claims volume (resulting in the elimination of 17 states).

DQ Atlas Standards for Enrollment Benchmarks and Claims Volume

|  | **Enrollment Benchmarks** | **Claims Volume** | |
| --- | --- | --- | --- |
|  | Average Monthly % Diff in Medicaid & CHIP Enrollment in TAF vs PI Data | Total header volume and total line volume as a percentage of the national median | Average number of line records per header as a percentage of the national median |
| Low concern | x ≤ 10% | 75% ≤ x ≤ 150% | 50% ≤ x ≤ 200% |
| Medium concern | 10% < x ≤ 20% | 50% ≤ x < 75%  or  150% < x ≤ 200% | 50% ≤ x ≤ 200% |
| High concern | 20% < x ≤ 50% | 10% ≤ x < 50%  or  x > 200% | 10% ≤ x < 50%  or  x > 200% |
| Unusable | x > 50% | x < 10% | x < 10% |
| Unclassified | NA | NA | NA |

**Part 2: Utilization measures**

For our main analysis, we focused on acute care visits as our primary outcome. Therefore, for each state, we assessed the percentage of all claims in each file that were missing the required fields for our analysis (outlined below). While DQ Atlas provides metrics on the missingness for each field, it does not provide metrics on whether a given claim is missing at least one key field. Therefore, in accordance with the DQ Atlas benchmarks, for each file, we required that at least 80% of all claim lines were not missing key fields for a given state (resulting in a final sample of 26 states and Washington DC).

Required fields for measuring healthcare utilization

| **File** | **Required Fields** |
| --- | --- |
| Other Services | Primary diagnosis, state, procedure code, service start date, service end date, and at least 1 measure of clinician specialty (NPI or CMS clinician specialty code) |
| Inpatient | Primary diagnosis, service start date, service end date |
| Pharmacy | Prescription fill date, National Drug Code (NDC), brand vs. generic indicator, and days supply |
| Long-term care | Primary diagnosis, service start date, service end date |

**Part 3: Spending measures**

For our secondary analysis, we used spending as the outcome measure. According to the Centers of Medicare and Medicaid, it is possible that a given state provided utilization measures (procedure code, diagnosis) for claims data, but did not report spending data for those claims.^2^ Further, even among states consistently reporting spending data, there is variability in the accuracy of their spending data.^2^

Therefore, among the 26 states and Washington DC that consistently reported utilization data, we identified the subset of states that reported accurate spending data from 2017-2019.

To identify states reporting quality spending data, we performed the following procedure for each state. Our spending measure focused on managed care and FFS encounters.

1. First, for each state-year, we separately assessed the percentage of MC claims and FFS claims missing spending information [Medicaid paid amount for FFS claims and line Medicaid FFS equivalent amount for MC claims; see **Methods** **Table 3** below for specific fields].
   1. We excluded states where > 10% of FFS or MC claims were missing spending information.
2. Second, for each state-year, we measured the total spending for MC claims and FFS claims. We also separately measured the total number of claim lines for MC and FFS claims.
   1. We excluded states where total spending for MC claims was approximately zero and total number of claim line items for MC claims were non-trivial. These were states where utilization data was provided, yet spending data was not populated for those claims.
   2. We included states where total spending for MC claims was approximately zero and the total number of claim line items for MC claims was approximately zero. These were states with no managed care members. As additional verification, we validated that these states, from 2017-2019, had no managed care programs using data from the Kaiser Family Foundation Medicaid Managed Care Tracker^3^– i.e., all patients receiving Medicaid were FFS members.
3. Third, for each state-year, we measured the spending per line claim lines for MC claims and FFS claims (measured separately for MC and FFS claims by dividing total spending by total number of claim lines). We assume, because spending for MC claims uses the FFS estimated spending, the spending per line for MC claims versus FFS claims should be similar. We excluded states where the absolute difference for spending per line item for MC vs. FFS claims was >$50 or the percentage difference was >20% (measured by FFS spending per line minus MC spending per line divided by FFS spending per line). We included a measure of absolute difference of >$50 because the spending per line for outpatient visits and medications were lower compared to hospitalizations and a difference of $30-50 could result in >20% in the percentage difference.

This resulted in a final sample of 6 states for our secondary cost-focused analysis: Alabama, Maine, Montana, Vermont, Wyoming, Illinois.

Fields for measuring spending

| **Claims file** | **Claim type** | **TAF Field** |
| --- | --- | --- |
| Inpatient file | Fee-for-service | MDCD_PD_AMT |
|  | Managed care | LINE_MDCD_FFS_EQUIV_AMT |
| Other services file | Fee-for-service | LINE_MDCD_PD_AMT |
|  | Managed care | LINE_MDCD_FFS_EQUIV_AMT |
| Pharmacy File | Fee-for-service | LINE_MDCD_PD_AMT |
|  | Managed care | LINE_MDCD_FFS_EQUIV_AMT |
| Long-term Service | Fee-for-service | MDCD_PD_AMT |
|  | Managed care | LINE_MDCD_FFS_EQUIV_AMT |

1. **Exclusion Criteria**

First, we excluded members residing in states that did not meet our data quality standards. Second, we eliminated members enrolled in two states in a given year, as it raised concerns regarding their enrollment status in Medicaid. Finally, we eliminated members who were dually enrolled in Medicare and Medicaid. Our final sample for the main analysis consisted of 30,619,475 members residing in 26 states and Washington DC.

Our analysis excluded dually eligible patients as Medicare is the primary source of health insurance coverage for these patients, and Medicaid, jointly funded by federal and state governments, provides supplemental coverage.^4,5^ Medicare covers the majority of medical services for dually enrolled patients, including inpatient and outpatient care, physician services, diagnostic and preventive care and, since 2006, outpatient prescription drugs.^4,5^ Therefore, the medical and pharmacy claims data required for our risk model for dually eligible patients would be found in Medicare claims data and not in TAF data. Additionally, Medicare patients typically have their own care management programs separate from Medicaid care management, where the former focus on elder care needs (including, e.g., hospice programs, in-home versus skilled nursing assessments, and readmission reductions for orthopedic and cardiovascular care) versus the typical primary care and social service focus of Medicaid care management programs (which include, e.g., a substantial focus on pediatric care gaps and prenatal care).

1. **Distribution of final sample by state**

We reported the percentage of total members in our final sample by state. Denominator is the final sample size (n=30,619,475). We order in descending order – i.e., largest or lowest fraction of members.

| State | Total number of members | Percentage of total members in final sample (n=30,619,475) |
| --- | --- | --- |
| Illinois | 3,334,144 | 10.9% |
| Pennsylvania | 3,245,429 | 10.6% |
| Michigan | 2,881,483 | 9.4% |
| Arizona | 2,216,416 | 7.2% |
| Washington | 2,144,674 | 7.0% |
| Indiana | 1,762,141 | 5.8% |
| Louisiana | 1,712,057 | 5.6% |
| Tennessee | 1,626,584 | 5.3% |
| Maryland | 1,558,895 | 5.1% |
| Kentucky | 1,496,598 | 4.9% |
| Virginia | 1,305,280 | 4.3% |
| Alabama | 1,110,457 | 3.6% |
| New Mexico | 960,835 | 3.1% |
| Nevada | 859,620 | 2.8% |
| Mississippi | 704,935 | 2.3% |
| West Virginia | 617,706 | 2.0% |
| Kansas | 450,641 | 1.5% |
| Utah | 438,798 | 1.4% |
| Hawaii | 394,120 | 1.3% |
| Idaho | 326,078 | 1.1% |
| Montana | 293,784 | 1.0% |
| Delaware | 276,482 | 0.9% |
| Washington DC | 262,232 | 0.9% |
| Maine | 249,484 | 0.8% |
| Vermont | 185,184 | 0.6% |
| North Dakota | 123,692 | 0.4% |
| Wyoming | 81,726 | 0.3% |

1. **Defining acute care visits**

**Identifying acute care visits**

We defined ED visits through Current Procedural Terminology codes (99281–85), revenue codes (0450–59, and 0981), or place-of-service codes (23). These visits were identified in the other services file.

According to the Centers of Medicare and Medicaid,^1^ the inpatient file captures all acute care hospitalizations–those that do and do not result from an ED visit. We include all hospitalizations in the inpatient file. Furthermore, for some states, there is a set of inpatient hospitalizations in the Other Services file. We identify these visits via the place of service code indicating inpatient care, diagnosis codes for childbirth, and procedure codes indicating inpatient care. We flag those visits as acute care hospitalizations.

**Defining non-emergent ED visits**

Non-emergent ED visits were identified by a Patched version of the New York University (NYU) algorithm, which was reported to have higher precision and clinical utility than the original NYU algorithm.^6^ Consistent with prior research,^7^ we defined a “non-emergent” as an ED visit with a principal diagnosis for a condition designated as non-emergent, primary care treatable, or preventable. Per the patched NYU algorithm, mental illness and substance abuse are not defined as non-emergent.

Furthermore, it is important to note that the NYU patch algorithm does not rely on ICD10 External Cause Codes (defined as ICD-10-CM codes beginning with a V, W, X, or Y). ICD10 External Cause Codes are secondary codes that capture specific details about an injury or health events. Prior research has found that many states underreport external cause codes in TAF data.^8^ However, because the NYU patch algorithm does not rely on ICD10 external cause codes, we believe the algorithm to be effective in identifying non-emergent ED visits.

For each ICD10 code, the NYU algorithm assigns a probability for its likelihood to be non-emergent, primary care treatable, or preventable. First, as per our definition of “non-emergent,” we summed the three probabilities. Second, we examined various probability cutoffs in **Methods Table 4** to evaluate their impact on the volume of ICD10 diagnoses considered as “non-emergent.” In accordance with prior research,^7^ we defined non-emergent ED visits as an ICD10 diagnosis with a 50% or higher likelihood of being non-emergent.

NYU ED Algorithm

####
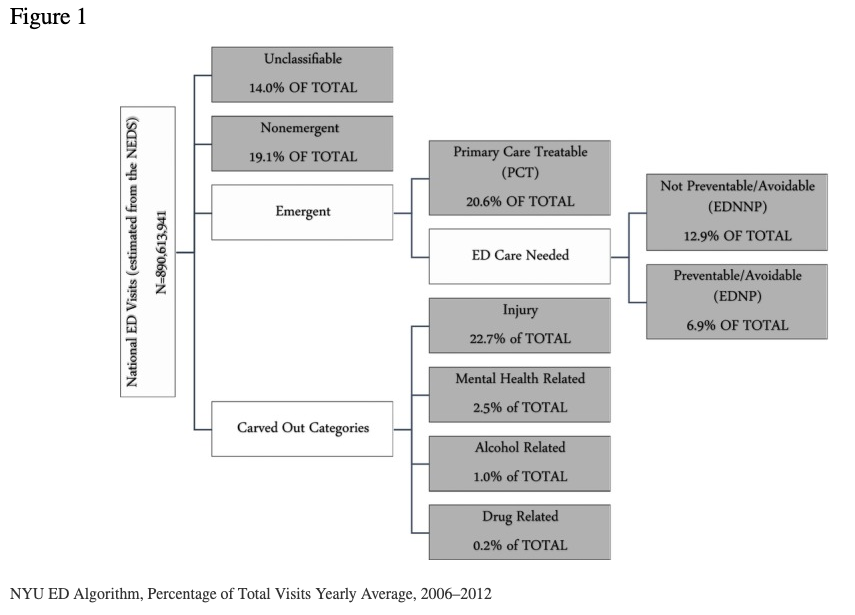


Johnston KJ, Allen L, Melanson TA, Pitts SR. A "Patch" to the NYU Emergency Department Visit Algorithm. *Health Serv Res.* 2017;52(4):1264-1276. doi:10.1111/1475-6773.1263

Percentage of non-emergent ICD10 diagnosis by probability cutoffs

| Probability cutoff | Percentage of non-emergent ICD10 diagnosis by probability cutoffs |
| --- | --- |
| >0% | 15.7% |
| 50% or greater | 15.1% |
| 75% or greater | 14.4% |
| 90% or greater | 14.0% |
| 100% | 13.8% |

**Defining non-emergent hospitalizations**

Non-emergent hospitalizations were identified by the Agency for Healthcare Research and Quality (AHRQ) Prevention Quality Indicator (PQI) algorithms.^9^ A number of the PQI non-emergent hospitalization measures have age-related restrictions. For example, Prevention Quality Indicator 01 (PQI 01) Diabetes Short-Term Complications Admission Rate encompasses ICD10 diagnoses of E1010, E1011, E10641, E1065, E1100, E1101, E11641, E1165 and is applicable to patients 18 and older. We ensured the age restrictions were applied accordingly.

**Defining episodes of care**

Next, to identify episodes of care, we linked ED and inpatient claims for the same patient if their dates of service overlapped or immediately followed one another. This resulted in “episodes of care” that included multiple claims for ED visits and/or hospitalizations. To address this, we implemented three strategies:

- First, under the assumption that a claim for a hospitalization would have a more accurate diagnosis versus a claim for an ED visit, in instances where an ED visits resulted in a hospitalization, and the primary diagnosis on the ED visit claim was identified as non-emergent by NYU patch, but the primary diagnosis on the hospitalization claim was identified as an emergency condition per PQI, we prioritized the diagnosis on the hospitalization, and defined the acute care episode as requiring emergent care.
- Second, in instances where there were multiple claims for ED visits for a given episode, if at least 1 claim in the episode had a diagnosis for an emergency condition per NYU patch, we defined the episode as requiring emergency care.
- Third, in instances where there were multiple claims for acute care inpatient visits (hospitalizations), if at least 1 claim in the episode had a diagnosis for an emergency condition per PQI, we defined the episode as requiring emergency care.

In summary, our approach was to ensure our analysis did not overestimate rates of non-emergent acute care use.

1. **Defining clinician specialty**

While there is high variability across states reporting clinician NPI and specialty,^1^ in our sample of 26 states and Washington DC, roughly 96.5% of claims had a valid servicing or billing clinician NPI. Our analysis prioritized the servicing clinician NPI, as we assumed this clinician delivered the care. However, for claims where the servicing clinician NPI was not provided, we used the billing clinician NPI. We derived clinician specialty via the CMS specialty code associated with the servicing or billing NPI on the claim. However, for claims where the clinician specialty code was not provided (which was the case for roughly 25% of claim lines), we derived it based on the taxonomy code, which was present for all clinicians in the Medicaid clinicians in the TAF Annual Provider Files.

We considered using MD-PPAS, a dataset that captures Medicare clinicians, to derive the clinician specialty code, as it is derived from Provider, Enrollment, Chain, and Ownership System (PECOS), which is known to be updated regularly. However, PECOS is a Medicare enrollment management system, and does not capture all Medicaid clinicians (there is roughly a 50% overlap between all servicing and billing clinician NPIs in TAF and MD-PPAS).^10^ Thus, we used the TAF Annual Provider Files as the source of truth for clinician specialty.

1. **Measures of area-level social determinants of health (SDOH)**

We included 13 county-level SDOH predictors from the AHRQ SDOH database.^11^ For patients residing in counties for which there were missing area-level SDOH measures, we imputed the mean based on their state of residence.

|  | **SDOH Feature from AHRQ data (data source*)** | **% missing for a given county in our data** |
| --- | --- | --- |
| Health care resources | Total number of substance abuse services facilities accepting Medicaid per 1,000 population (AMFAR data) | 0.28% |
|  | Total number of substance abuse treatment facilities offering all three medication assisted treatment services that accept Medicaid per 1,000 population (AMFAR data) | 0.28% |
|  | Total number of facilities that provide mental health services and accept Medicaid per 1,000 population (AMFAR data) | 0.28% |
|  | Total number of APRNs/PAs with NPI per 1,000 population (AHRF data) | 0.25% |
|  | Total number of urgent care organizations per 1,000 population (AHRF data) | 0.06% |
| Social conditions | Population density (proxy for rurality) (US Census data) | 0.25% |
|  | Percentage of population with income to poverty ratio under 0.50 (ACS data) | 0.25% |
|  | Percentage of households with public assistance income or food stamps/SNAP (ACS data) | 0.25% |
|  | Percentage of population with less than high school education (ages 25 and over) (ACS data) | 0.25% |
| Death rates | Total number of deaths from injury per 100,000 population (CDC WONDER data) | 6.9% |
|  | Total number of drug overdose deaths involving any opioid per 100,000 population (CDC WONDER data) | 7.2% |
| Environmental factors | Percentage of days with good air quality (AHRF data) | 8.9% |
|  | Total number of days with daily maximum heat index, absolute threshold: 100°F (NEPHTN data) | 3.7% |

Abbreviations: Area Health Resources Files (AHRF); CDC (Centers for Disease Control); The American foundations for AIDS Research (AMFAR); American Community Survey (ACS); National Environmental Public Health Tracking Network (NEPHTN)

1. **Person-level Measures of Social Determinants of Health**

As described in Table 1 of the manuscript, there is wide variation in missingness among the person-level demographic and SDOH predictor variables, ranging from 0.8% for age category to 85.3% for speaking English.

Due to variation in missing data for person-level characteristics, we included a missing category for each characteristic instead of imputing missing data. This approach acknowledges that the presence of missingness itself may provide valuable information for model fitting.

| Characteristic Group | Characteristic | N (col %) |
| --- | --- | --- |
| Age Category | Missing | 249,738 (0.8) |
| Sex | Missing | 395,197 (1.3) |
| Race/ Ethnicity | Missing | 7,450,299 (24.3) |
| Household Size | Missing | 14,202,814 (46.4) |
| Federal Poverty Line | Missing | 14,185,091 (46.3) |
| English Speaking | Missing | 26,112,225 (85.3) |
| Married | Missing | 11,993,722 (39.2) |
| US Citizen | Missing | 3,985,106 (13.0) |
| Receipt of SSI | Missing | 2,278,510 (7.4) |
| Receipt of SSDI | Missing | 13,194,731 (43.1) |
| Receipt of TANF | Missing | 7,351,598 (24.0) |

1. **Defining members with a disability**

We defined members as having a disability if the Demographic and Eligibility file or Disability and Need Supplemental File indicated they had any disability.

1. **Prediction and Outcome Measurement Periods**

Our analysis identified the first month a member was enrolled in Medicaid from 2017-2018. We focused on 2017-2018 because we measured the 12-month period following the first month of enrollment. The first month (indicated in black) was considered a wash period, as members may not have enrolled in Medicaid on the first day of the month. The predictor measurement period was the six-month period following the first month of enrollment (indicated in red). The outcome measurement period (indicated in blue) was the six-month period following the prediction measurement period.

| Jan 2017 | Feb 2017 | Mar 2017 | Apr 2017 | May 2017 | Jun 2017 | Jul 2017 | Aug 2017 | Sep 2017 | Oct 2017 | Nov 2017 | Dec  2017 | Jan  2018 | Feb  2018 | Mar  2018 | Apr  2018 | May  2018 | Jun  2018 | Jul  2018 | Aug  2018 | Sep  2018 | Oct  2018 | Nov  2018 | Dec  2018 | Jan  2019 | Feb  2019 | Mar  2019 | Apr  2019 | May  2019 | Jun  2019 | Jul  2019 | Aug  2019 | Sep  2019 | Oct  2019 | Nov 2019 | Dec  2019 |
| --- | --- | --- | --- | --- | --- | --- | --- | --- | --- | --- | --- | --- | --- | --- | --- | --- | --- | --- | --- | --- | --- | --- | --- | --- | --- | --- | --- | --- | --- | --- | --- | --- | --- | --- | --- |
| x | x | x | x | x | x | x | x | x | x | x | x | x |  |  |  |  |  |  |  |  |  |  |  |  |  |  |  |  |  |  |  |  |  |  |  |
|  | x | x | x | x | x | x | x | x | x | x | x | x | x |  |  |  |  |  |  |  |  |  |  |  |  |  |  |  |  |  |  |  |  |  |  |
|  |  | x | x | x | x | x | x | x | x | x | x | x | x | x |  |  |  |  |  |  |  |  |  |  |  |  |  |  |  |  |  |  |  |  |  |
|  |  |  | x | x | x | x | x | x | x | x | x | x | x | x | x |  |  |  |  |  |  |  |  |  |  |  |  |  |  |  |  |  |  |  |  |
|  |  |  |  | x | x | x | x | x | x | x | x | x | x | x | x | x |  |  |  |  |  |  |  |  |  |  |  |  |  |  |  |  |  |  |  |
|  |  |  |  |  | x | x | x | x | x | x | x | x | x | x | x | x | x |  |  |  |  |  |  |  |  |  |  |  |  |  |  |  |  |  |  |
|  |  |  |  |  |  | x | x | x | x | x | x | x | x | x | x | x | x | x |  |  |  |  |  |  |  |  |  |  |  |  |  |  |  |  |  |
|  |  |  |  |  |  |  | x | x | x | x | x | x | x | x | x | x | x | x | x |  |  |  |  |  |  |  |  |  |  |  |  |  |  |  |  |
|  |  |  |  |  |  |  |  | x | x | x | x | x | x | x | x | x | x | x | x | x |  |  |  |  |  |  |  |  |  |  |  |  |  |  |  |
|  |  |  |  |  |  |  |  |  | x | x | x | x | x | x | x | x | x | x | x | x | x |  |  |  |  |  |  |  |  |  |  |  |  |  |  |
|  |  |  |  |  |  |  |  |  |  | x | x | x | x | x | x | x | x | x | x | x | x | x |  |  |  |  |  |  |  |  |  |  |  |  |  |
|  |  |  |  |  |  |  |  |  |  |  | x | x | x | x | x | x | x | x | x | x | x | x | x |  |  |  |  |  |  |  |  |  |  |  |  |
|  |  |  |  |  |  |  |  |  |  |  |  | x | x | x | x | x | x | x | x | x | x | x | x | x |  |  |  |  |  |  |  |  |  |  |  |
|  |  |  |  |  |  |  |  |  |  |  |  |  | x | x | x | x | x | x | x | x | x | x | x | x | x |  |  |  |  |  |  |  |  |  |  |
|  |  |  |  |  |  |  |  |  |  |  |  |  |  | x | x | x | x | x | x | x | x | x | x | x | x | x |  |  |  |  |  |  |  |  |  |
|  |  |  |  |  |  |  |  |  |  |  |  |  |  |  | x | x | x | x | x | x | x | x | x | x | x | x | x |  |  |  |  |  |  |  |  |
|  |  |  |  |  |  |  |  |  |  |  |  |  |  |  |  | x | x | x | x | x | x | x | x | x | x | x | x | x |  |  |  |  |  |  |  |
|  |  |  |  |  |  |  |  |  |  |  |  |  |  |  |  |  | x | x | x | x | x | x | x | x | x | x | x | x | x |  |  |  |  |  |  |
|  |  |  |  |  |  |  |  |  |  |  |  |  |  |  |  |  |  | x | x | x | x | x | x | x | x | x | x | x | x | x |  |  |  |  |  |
|  |  |  |  |  |  |  |  |  |  |  |  |  |  |  |  |  |  |  | x | x | x | x | x | x | x | x | x | x | x | x | x |  |  |  |  |
|  |  |  |  |  |  |  |  |  |  |  |  |  |  |  |  |  |  |  |  | x | x | x | x | x | x | x | x | x | x | x | x | x |  |  |  |
|  |  |  |  |  |  |  |  |  |  |  |  |  |  |  |  |  |  |  |  |  | x | x | x | x | x | x | x | x | x | x | x | x | x |  |  |
|  |  |  |  |  |  |  |  |  |  |  |  |  |  |  |  |  |  |  |  |  |  | x | x | x | x | x | x | x | x | x | x | x | x | x |  |
|  |  |  |  |  |  |  |  |  |  |  |  |  |  |  |  |  |  |  |  |  |  |  | x | x | x | x | x | x | x | x | x | x | x | x | x |

1. **Defining two-stage analysis**

**Overview**

**

**

Given the high disenrollment rates in Medicaid,^12^ we conducted a two-stage analysis. In the first stage, we predicted the likelihood a member would lose their Medicaid coverage in the one-year period after their first month of enrollment in Medicaid from 2017-2018. In the second stage, for each member in the stage 2 sample, we predicted the probability of losing Medicaid coverage in the one-year period following enrollment in Medicaid. We included the probability of losing Medicaid coverage as a predictor in the stage 2 model, in addition to the other predictors described in the manuscript.

We explored imposing a continuous enrollment period of one year. However, dissimilar to Medicare beneficiaries, Medicaid members often churn in and out of the Medicaid program. Roughly 25% of the 30.6M Medicaid members in our sample lost their Medicaid coverage in the one-year period following their first month of enrollment, with wide variation across states (ranging from 9% in Kentucky to 45% in Utah; **Supplement Tables 2-3**).

Thus, we incorporated the likelihood that a given Medicaid member would lose their Medicaid coverage in the one-year period following their initial month of coverage in our risk model. Imposing an arbitrary cut point of continuous eligibility would reduce generalizability of our model.

**Stage 1 Analysis**

First, we randomly split our sample of 30.6M patients into two samples of roughly 15.3M patients. Second, using the stage 1 sample of 15.3 patients, we predicted the probability that a member would lose their Medicaid coverage in the one-year period after their first month of enrollment. We explored various models – logistic regression, regularized logistic regression, and XGBoost – and selected the highest performing model as the final model. Finally, using the highest performing model, for each member in the stage 2 sample, we predicted their probability of losing Medicaid coverage (results can be found in **Supplement Tables 4-5**). The predicted probability was used as a feature for all stage 2 models.

**Stage 2 Analysis**

Stage 2 analysis predicted the likelihood each member would use acute care services. Their probability of losing Medicaid coverage was used as a predictor in addition to the others outlined in the manuscript. Stage 2 models included the ‘Baseline Comparison’, ‘Cumulative Risk and Risk Trajectories Measures’, ‘Area SDOH’, and ‘Area and Individual SDOH’ models. Each of these models (i.e., ‘Baseline Comparison’ to ‘Area and Individual SDOH’) includes a patient’s probability of losing coverage within 12 months as a predictor, which was predicted as part of the first stage analysis. The first six-month period after enrollment was the predictor measurement period; the second six-month period was the outcome measurement period;

1. **Model Specification**

**Stage 1 Model**

| **Outcome vs. predictor** | **Type** | **Measure** | **Description** |
| --- | --- | --- | --- |
| Outcome measures | Medicaid coverage | Loss of Medicaid coverage | Binary indicator (yes/no) for whether a patient loses their Medicaid coverage in the 12-month period following enrollment |
| Predictors | Patient demographics and individual-level SDOH measures | Age (continuous) | Measured as years |
|  |  | Sex (categorical) | Male, female, missing |
|  |  | Race/ethnicity (categorical) | White, Black, Hispanice, Asian, Native American, missing |
|  |  | House size (categorical) | Single, 2-5, 6 or more, missing |
|  |  | Income level (categorical) | Modified to 0-100% federal poverty level, 100-200%, 200% or more, missing |
|  |  | English speaking (categorical) | yes/no/missing |
|  |  | Married (categorical) | yes/no/missing |
|  |  | US citizen (categorical) | yes/no/missing |
|  |  | Receipt of supplemental security income (SSI) (categorical) | yes/no/missing |
|  |  | Receipt of social security disability insurance (SSDI) (categorical) | yes/no/missing |
|  |  | Receipt of temporary assistance for needy families (TANF) (categorical) | yes/no/missing |
|  |  | Disabled (categorical) | yes/no/missing |
|  | Medicaid  coverage | Month of enrollment (categorical) | Month of year patient enrolled in Medicaid (january-december) |
|  |  | Year of enrollment (categorical) | Year patient enrolled in Medicaid (2017, 2018) |
|  |  | State (categorical) | State patient was enrolled in Medicaid |
|  |  | Medicaid coverage in 2016 (categorical) | Binary indicator for whether patient was enrolled in Medicaid in 2016 |

**Stage 2 Model**

| **Outcome vs. predictor** | **Type** | **Measure** | **Description** |
| --- | --- | --- | --- |
| Outcome measures | Acute care utilization | At least 1 all-cause ED visit or hospitalization during 6-month outcome measurement period | Binary indicator (yes/no) |
|  |  |  |  |
|  |  | At least 1 non-emergent ED visit or hospitalization during 6-month outcome measurement period | Binary indicator (yes/no) |
| Predictors | Loss of Medicaid Coverage | Probability value between 0-1 for likelihood of losing coverage within a year | Scaled using standard scaler |
|  | Fixed effect | Month of enrollment (categorical) | Month patient enrolled in Medicaid |
|  |  | Year of enrollment (categorical) | Year patient enrolled in Medicaid (2017, 2018) |
|  |  | State (categorical) | Patient state of residence |
|  | Demographics | Age (continuous) | Measured as years |
|  |  | Sex (categorical) | Male, female, missing |
|  |  | Race/ethnicity (categorical) | White, Black, Hispanice, Asian, Native American, missing |
|  |  | Disabled (categorical) | yes/no/missing |
|  | Clinical History | Clinical condition (continuous) | Scaled using standard scaler |
|  |  | Type of care (continuous) | Scaled using standard scaler |
|  |  | Medication type (continuous) | Scaled using standard scaler |
|  |  | Clinician type (continuous) | Scaled using standard scaler |
|  | Cumulative risk and risk trajectories | Slope of all-cause ED visits and hospitalizations over 6 month period (continuous) | Scaled using standard scaler |
|  |  | Slope of all-cause ED visits and hospitalizations over 6 month period (continuous) | Scaled using standard scaler |
|  |  | Slope of total number of medication prescriptions over 6 month period (continuous) | Scaled using standard scaler |
|  |  | Total number of days for hospitalizations during 6 month period (continuous) | Scaled using standard scaler |
|  |  | Total number of all cause ED visits and hospitalizations over 6 month period (continuous) | Scaled using standard scaler |
|  |  | Percentage of all ED visits & hospitalizations for non-emergent conditions over 6 month period (continuous) | Scaled using standard scaler |
|  |  | Total days supply of medication across all medications (continuous) | Scaled using standard scaler |
|  |  | Total number of unique medications (continuous) | Scaled using standard scaler |
|  |  | Total number of medication prescriptions (continuous) | Scaled using standard scaler |
|  |  | Percentage of prescriptions for generic medications (continuous) | Scaled using standard scaler |
|  |  | Probability of losing Medicaid coverage | Scaled using standard scaler |
|  | Area SDOH measures  (continuous measures) | Total number of substance abuse services facilities accepting Medicaid per 1,000 population (AMFAR) | Scaled using standard scaler |
|  |  | Total number of substance abuse treatment facilities offering all three medication assisted treatment services that accept Medicaid per 1,000 population (AMFAR) | Scaled using standard scaler |
|  |  | Total number of facilities that provide mental health services and accept Medicaid per 1,000 population (AMFAR) | Scaled using standard scaler |
|  |  | Population density (proxy for rurality) (US Census) | Scaled using standard scaler |
|  |  | Percentage of population with income to poverty ratio under 0.50 (ACS) | Scaled using standard scaler |
|  |  | Percentage of households with public assistance income or food stamps/SNAP (ACS) | Scaled using standard scaler |
|  |  | Percentage of population with less than high school education (ages 25 and over) (ACS data) | Scaled using standard scaler |
|  |  | Total number of deaths from injury per 100,000 population (CDC WONDER) | Scaled using standard scaler |
|  |  | Total number of drug overdose deaths involving any opioid per 100,000 population (CDC WONDER) | Scaled using standard scaler |
|  |  | Total number of APRNs/PAs with NPI per 1,000 population (AHRF)* | Scaled using standard scaler |
|  |  | Total number of urgent care organizations per 1,000 population (AHRF) | Scaled using standard scaler |
|  |  | Percentage of days with good air quality (AHRF) | Scaled using standard scaler |
|  |  | Total number of days with daily maximum heat index, absolute threshold: 100°F (NEPHTN) | Scaled using standard scaler |
|  | Individual SDOH measures | House size (categorical) | Single, 2-5, 6 or more, missing |
|  |  | Income level (categorical) | Modified to 0-100% federal poverty level, 100-200%, 200% or more, missing |
|  |  | English speaking (categorical) | yes/no/missing |
|  |  | Married (categorical) | yes/no/missing |
|  |  | US citizen (categorical) | yes/no/missing |
|  |  | Receipt of supplemental security income (SSI) (categorical) | yes/no/missing |
|  |  | Receipt of social security disability insurance (SSDI) (categorical) | yes/no/missing |
|  |  | Receipt of temporary assistance for needy families (TANF) (categorical) | yes/no/missing |

*APRNs defined as nurse practitioners, clinical nurse specialists, and advanced practice nurses

1. **Machine learning procedure**

**Train, Validate, and Test Sample**

test cohort

(20% of stage 1 sample pf 5M)

training cohort

(80% of stage 1 sample of 5M)

Stage 1 Sample

(n=15,309,738)

Final Sample

(n=30,619,475)

training cohort

(80% of stage 2 sample of 5M)

test cohort

(20% of stage 2 sample pf 5M)

First month of enrollment in Medicaid in 2017-18

Randomly selected 5M patients due to limited compute capacity in CMS VRDC environment

Random sample of 5 million

Random sample of 5 million

validation

(20% of training cohort of 4M)

training

(80% of training cohort of 4M)

validation

(20% of training cohort of 4M)

training

(80% of training cohort of 4M)

Stage 2 Sample

(n=15,309,737)

First, in both stages, we selected a random sample of roughly 5 million patients out of the 15.3 million (roughly 33% of the sample) due to the limited compute capacity in the Chronic Conditions Warehouse (CCW) Virtual Research Data Center (VRDC).

Using the random sample of 5 million patients, we then randomly divided the study sample into a training (80 percent of patients) and testing cohort (20 percent of patients). The training cohort was used to build the model, whereas the testing cohort was used to evaluate the accuracy of the model. Next, the training cohort was randomly partitioned into two subsets (consisting of 80 percent and 20 percent of patients in the training cohort). We used a simple hold-out validation approach versus k-fold cross validation to ensure model convergence. We used the hold-out test sample to identify the best tuning parameters for the regularized regression and XGBoost. Specifically, we set up a grid for each combination of parameters, applied the algorithm with those parameters to 80 percent of the development set, and assessed the performance of the solution on 20 percent. This procedure was for each parameter set in the grid, and the optimal parameters were selected according to a maximum C-statistic.

For each machine learning algorithm, a final model was obtained by retraining the algorithm on the entire training set (80% of the sample) using the optimal parameters selected, and the performance of this final model was assessed on the test cohort (20% of the sample).

**Point Estimate and 95% CI Intervals:**

We used a common method to estimate the 95% confidence intervals for performance measures.^13,14^ The final model is selected based on the point estimate method. Bootstrapping is then used to estimate the 95% confidence intervals for our performance metrics using the test data:

1. **Bootstrap Resampling for Test Data:** We implemented a bootstrapping procedure that involved repeatedly drawing random samples with replacement from the full test dataset. For each bootstrapped sample, we measured each evaluation metric (AUC, MCC, F1 Score, PPV, NPV, R-squared, accuracy, sensitivity, specificity).
2. **Confidence Intervals for Training Metrics:** After obtaining the metrics for each bootstrap sample, we calculated the mean and 95% confidence intervals for each metric..

**Hyperparameters**

The following hyperparameters were used to find the optimal hyperparameters for each regularized logistic regression and XGBoost model.

|  | **Hyperparameter GridSearch** |
| --- | --- |
| Regularized logistic regression  [acute care visits] | regParam: 0.0001, 0.001, 0.01, 0.1, 1.0, 10.0, 100.0  elasticNetParam: 0, 0.2, 0.4, 0.6, 0.8, 1.0 |
| Random Forest  [acute care visits] | minInstancesPerNode: [1, 10, 20, 30, 40, 50, 60]  featureSubsetStrategy: [“auto”, “sqrt”, “log2”]  Maxdepth: [10, 12, 14, 16, 18, 20]  numTrees: [10, 20, 50, 75, 100, 125, 150, 175, 200] |
| XGBoost  [acute care visits and cost] | Learning rate: 0.005, 0.01, 0.02, 0.05  Subsample: 0.2, 0.3, 0.4, 0.5, 0.6, 0.7, 0.8, 0.9, 1.0  Min_child_weight: 0, 0.5, 1, 2, 3, 4, 5  Colsample_bytree: 0.2, 0.3, 0.4, 0.5, 0.6, 0.7, 0.8, 0.9, 1.0  Max_depth: 10, 12, 14, 16, 18, 20, 22, 24  Reg_alpha: 0.0, 0.25, 0.5, 0.75, 1.0  Reg_lambda: 0.0, 0.25, 0.5, 0.75, 1.0  Gamma: 0.0, 0.25, 0.5, 0.75, 1.0  Colsample_bylevel: 0.2, 0.3, 0.4, 0.5, 0.6, 0.7, 0.8, 0.9, 1.0 |

1. **Distribution of predictors and outcomes for total sample of versus random sample of patients**

**Distribution of Predictors:**

- For the distribution of predictors, we compare the total sample of 30.6M to the random sample of 10M patients.
- The distribution of predictor variables was identical with standardized mean differences of 0.00 for all predictors.

**
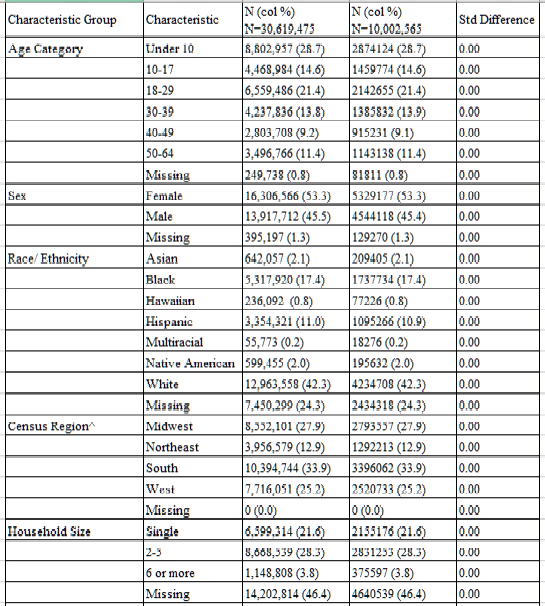
**

**
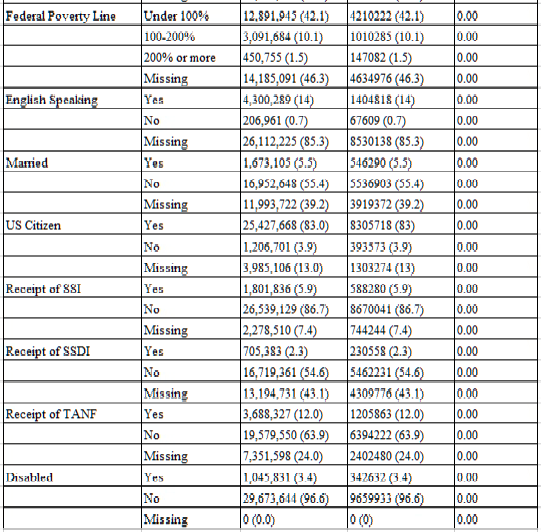
**

**Distribution of Outcomes:**

- Because the stage two analysis estimates acute care utilization, we compare the distribution of the stage two sample of 15.3M to the random sample of 5M patients used for our analysis.
- The distribution of outcome variables was nearly identical with standardized mean differences under 0.04 for both outcome variables.

**
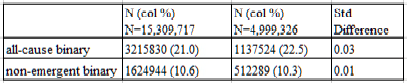
**

1. **Sensitivity Analyses**

We conducted the following sensitivity analyses using our highest performing model. First, we eliminated race/ethnicity from the model to understand the impact on the equity results. Our primary model included race/ethnicity because we believe these variables would best capture the impact of structural racism on likelihood of accessing preventive care, which we theorized as averting non emergent acute care. However, we wished to explicitly omit the variables to understand how their inclusion versus exclusion could influence our study of bias in acute care utilization across race/ethnic groups.

Second, given the large volume of children in our sample, we performed our analysis with adults only. We specifically recognized that most of the non-emergent ED visits and hospitalizations would be among adults, hence we wished to understand how inclusion versus exclusion of children would affect our estimation of model accuracy and precision.

Third, we evaluated the impact of down sampling White patients (effectively up sampling minority patients relative to White patients) to reduce underestimation for minority patients.

Fourth, because class imbalanced data hinders classification performance of RF, for the best performing RF model, we performed a down sampling procedure, specifically training on a disproportionately lower subset of patients with no acute care events.

**References:**

1. Medicaid.gov. DQ atlas [Internet]. Baltimore (MD): Centers for Medicare and Medicaid Services; [cited 2023 April 1]. Available from: https:// [www.medicaid.gov/dq-atlas/](http://www.medicaid.gov/dq-atlas/)
2. https://resdac.org/sites/datadocumentation.resdac.org/files/2021-08/TAF_TechGuide_Claims_Files.pdf
3. <https://www.kff.org/statedata/collection/medicaid-managed-care-tracker/>
4. <https://www.kff.org/report-section/a-primer-on-medicare-what-is-the-role-of-medicare-for-dual-eligible-beneficiaries/>
5. <https://www.kff.org/medicare/issue-brief/how-do-dual-eligible-individuals-get-their-medicare-coverage/>
6. Johnston KJ, Allen L, Melanson TA, Pitts SR. A "Patch" to the NYU Emergency Department Visit Algorithm. *Health Serv Res*. 2017;52(4):1264-1276. doi:10.1111/1475-6773.12638
7. Giannouchos TV, Ukert B, Andrews C. Association of Medicaid Expansion With Emergency Department Visits by Medical Urgency. *JAMA Netw Open*. 2022;5(6):e2216913. Published 2022 Jun 1. doi:10.1001/jamanetworkopen.2022.16913
8. Nguyen JK, Sanghavi P. A national assessment of legacy versus new generation Medicaid data. *Health Serv Res*. 2022;57(4):944-956. doi:10.1111/1475-6773.13937
9. Agency for Healthcare Research and Quality. Guide to Prevention Quality Indicators: Hospital Admission for Ambulatory Care Sensitive Conditions. Department of Health and Human Services; 2001. AHRQ publication no. 02-R0203.
10. <https://www.cms.gov/Medicare/Provider-Enrollment-and-Certification/Manage-Your-Enrollment#:~:text=PECOS%20is%20the%20online%20Medicare,Review%20and%20update%20your%20information>
11. Determinants of Health (SDOH) Data. Accessed [April 15, 2023]. <https://www.ahrq.gov/sdoh/data-analytics/sdoh-data.html#download>
12. https://www.macpac.gov/wp-content/uploads/2021/10/An-Updated-Look-at-Rates-of-Churn-and-Continuous-Coverage-in-Medicaid-and-CHIP.pdf
13. Efron B, Tibshirani RJ. An Introduction to the Bootstrap. Chapman & Hall/CRC; 1994.
14. Hastie T, Tibshirani R, Friedman J. The Elements of Statistical Learning: Data Mining, Inference, and Prediction. 2nd ed. Springer; 2009.

**Table 1:** Transparent reporting of a multivariable prediction model for individual prognosis or diagnosis (TRIPOD): The TRIPOD statement

| **Section/Topic** | **Item** |  | **Checklist Item** | **Page / Section** |
| --- | --- | --- | --- | --- |
| **Title and abstract** | | | | |
| Title | 1 | D;V | Identify the study as developing and/or validating a multivariable prediction model, the target population, and the outcome to be predicted. | 1 |
| Abstract | 2 | D;V | Provide a summary of objectives, study design, setting, participants, sample size, predictors, outcome, statistical analysis, results, and conclusions. | 2 |
| **Introduction** | | | | |
| Background and objectives | 3a | D;V | Explain the medical context (including whether diagnostic or prognostic) and rationale for developing or validating the multivariable prediction model, including references to existing models. | 4 |
|  | 3b | D;V | Specify the objectives, including whether the study describes the development or validation of the model or both. | 5 |
| **Methods** | | | | |
| Source of data | 4a | D;V | Describe the study design or source of data (e.g., randomized trial, cohort, or registry data), separately for the development and validation data sets, if applicable. | 7 |
|  | 4b | D;V | Specify the key study dates, including start of accrual; end of accrual; and, if applicable, end of follow-up. | 7 |
| Participants | 5a | D;V | Specify key elements of the study setting (e.g., primary care, secondary care, general population) including number and location of centers. | 8 |
|  | 5b | D;V | Describe eligibility criteria for participants. | 9 |
|  | 5c | D;V | Give details of treatments received, if relevant. | N/A |
| Outcome | 6a | D;V | Clearly define the outcome that is predicted by the prediction model, including how and when assessed. | 10 |
|  | 6b | D;V | Report any actions to blind assessment of the outcome to be predicted. | 10 |
| Predictors | 7a | D;V | Clearly define all predictors used in developing or validating the multivariable prediction model, including how and when they were measured. | 11-14 |
|  | 7b | D;V | Report any actions to blind assessment of predictors for the outcome and other predictors. | 11-14 |
| Sample size | 8 | D;V | Explain how the study size was arrived at. | 7-9 |
| Missing data | 9 | D;V | Describe how missing data were handled (e.g., complete-case analysis, single imputation, multiple imputation) with details of any imputation method. | 9, 13 |
| Statistical analysis methods | 10a | D | Describe how predictors were handled in the analyses. | 11-14 |
|  | 10b | D | Specify type of model, all model-building procedures (including any predictor selection), and method for internal validation. | 14-15 |
|  | 10c | V | For validation, describe how the predictions were calculated. | 14-15 |
|  | 10d | D;V | Specify all measures used to assess model performance and, if relevant, to compare multiple models. | 14-15 |
|  | 10e | V | Describe any model updating (e.g., recalibration) arising from the validation, if done. | 16-17 |
| Risk groups | 11 | D;V | Provide details on how risk groups were created, if done. | N/A |
| Development vs. validation | 12 | V | For validation, identify any differences from the development data in setting, eligibility criteria, outcome, and predictors. | 16-17 |
| **Results** | | | | |
| Participants | 13a | D;V | Describe the flow of participants through the study, including the number of participants with and without the outcome and, if applicable, a summary of the follow-up time. A diagram may be helpful. | Supplement  Figure 1 |
|  | 13b | D;V | Describe the characteristics of the participants (basic demographics, clinical features, available predictors), including the number of participants with missing data for predictors and outcome. | Table 1; Results Section |
|  | 13c | V | For validation, show a comparison with the development data of the distribution of important variables (demographics, predictors and outcome). | Supplement Tables 11-14 |
| Model development | 14a | D | Specify the number of participants and outcome events in each analysis. | Supplement Figure 1 |
|  | 14b | D | If done, report the unadjusted association between each candidate predictor and outcome. | N/A |
| Model specification | 15a | D | Present the full prediction model to allow predictions for individuals (i.e., all regression coefficients, and model intercept or baseline survival at a given time point). | Supplement Tables 11-14; 18-21 |
|  | 15b | D | Explain how to use the prediction model. | Methods section |
| Model performance | 16 | D;V | Report performance measures (with CIs) for the prediction model. | Results section |
| Model-updating | 17 | V | If done, report the results from any model updating (i.e., model specification, model performance). | N/A |
| **Discussion** | | | | |
| Limitations | 18 | D;V | Discuss any limitations of the study (such as non representative sample, few events per predictor, missing data). | Discussion Section |
| Interpretation | 19a | V | For validation, discuss the results with reference to performance in the development data, and any other validation data. | Discussion Section |
|  | 19b | D;V | Give an overall interpretation of the results, considering objectives, limitations, results from similar studies, and other relevant evidence. | Discussion Section |
| Implications | 20 | D;V | Discuss the potential clinical use of the model and implications for future research. | Discussion Section |
| **Other information** | | | | |
| Supplementary information | 21 | D;V | Provide information about the availability of supplementary resources, such as study protocol, Web calculator, and data sets. | Full Supplement |
| Funding | 22 | D;V | Give the source of funding and the role of the funders for the present study. | N/A |

**Table 2:** Total number of months enrolled in Medicaid in 12-month period after first month of enrollment from 2017-18

We reported the number of months patients in our final sample (n=30,619,475) who were enrolled in the 12-month period after their first month of enrollment from 2017-2018.

| Number of months | Number of patients | Percentage |
| --- | --- | --- |
| 0 | 426779 | 1.39 |
| 1 | 585903 | 1.91 |
| 2 | 478474 | 1.56 |
| 3 | 458953 | 1.5 |
| 4 | 498489 | 1.63 |
| 5 | 508081 | 1.66 |
| 6 | 521926 | 1.7 |
| 7 | 584707 | 1.91 |
| 8 | 573207 | 1.87 |
| 9 | 628886 | 2.05 |
| 10 | 700720 | 2.29 |
| 11 | 1568747 | 5.12 |
| 12 | 23084603 | 75.39 |

**Table 3:** Percentage of patients who lose Medicaid Coverage within 12-month period by State

| **State** | **Percentage of patient losing Medicaid coverage in 12-month period after first month of coverage from 2017-18** |
| --- | --- |
| Utah | 45.2% |
| Wyoming | 42.3% |
| North Dakota | 41.4% |
| Nevada | 39.2% |
| Delaware | 32.7% |
| Indiana | 32.3% |
| West Virginia | 31.9% |
| Pennsylvania | 31.2% |
| Kansas | 29.8% |
| Idaho | 28.0% |
| Washington | 27.6% |
| Maine | 26.9% |
| Mississippi | 26.3% |
| Michigan | 26.1% |
| Virginia | 24.4% |
| Arizona | 24.0% |
| Alabama | 23.8% |
| Vermont | 22.8% |
| New Mexico | 22.3% |
| Montana | 22.0% |
| Hawaii | 20.5% |
| Maryland | 20.2% |
| Illinois | 19.4% |
| Louisiana | 17.9% |
| Tennessee | 16.7% |
| Washington DC | 10.1% |
| Kentucky | 9.0% |

**Table 4:** Model Performance for Stage 1 analysis [predicting loss of Medicaid coverage]

| **Model and outcome** | **AUC**  **(95% CI)** | **Accuracy (95% CI)** | **MCC**  **(95% CI)** | **Sensitivity (95% CI)** | **Specificity**  **(95% CI)** | **NPV**  **(95% CI)** | **PPV**  **(95% CI)** |
| --- | --- | --- | --- | --- | --- | --- | --- |
| Logistic Regression | 0.694  (0.690, 0.701) | 0.769  (0.767, 0.773) | 0.211  (0.203, 0.224) | 0.114  (0.110, 0.121) | 0.983  (0.982, 0.984) | 0.772  (0.770, 0.777) | 0.688  (0.670, 0.709) |
| Reg. Logistic Regression | 0.695  (0.690, 0.701) | 0.770  (0.765, 0.773) | 0.212  (0.200, 0.221) | 0.115  (0.108, 0.120) | 0.983  (0.982, 0.984) | 0.773  (0.769, 0.776) | 0.688  (0.664, 0.706) |
| Random Forest | 0.739  (0.738, 0.743) | 0.783  (0.782, 0.785) | 0.293  (0.289, 0.297) | 0.170  (169, 0.175) | 0.983  (0.982, 0.984) | 0.784  (0.783, 0.786) | 0.766  (0.761, 0.776) |
| XGBoost | 0.749  (0.744, 0.753) | 0.786  (0.782, 0.789) | 0.309  (0.300, 0.319) | 0.209  (0.203, 0.217) | 0.975  (0.973, 0.976) | 0.790  (0.786, 0.794) | 0.724  (0.716, 0.744) |

Abbreviations: Area under the ROC Curve (AUC); Matthews correlation coefficient (MCC); Negative predictive value (NPV); Positive predictive value (PPV); Extreme gradient boosting (XGBoost)

*We compared three modeling approaches – logistic regression, regularized logistic regression, random forest, and XGBoost models. Models incorporate measures of patient demographics, coverage information, and person-level SDOH. See **Supplement Methods** for model specification.

**Table 5:** Importance scores for predicting loss of Medicaid coverage [all features]

| **Predictor** | **Logistic regression**  **[beta coefficient weights]** | **Logistic regression with regularization**  **[beta coefficient weights]** | **XGBoost feature importance**  **[based on importance type of gain]** | **XGBoost feature importance normalized from 0-100** |
| --- | --- | --- | --- | --- |
| state_KY | -1.831 | -1.679 | 491.402 | 100.0 |
| state_IL | -0.713 | -0.575 | 319.263 | 65.0 |
| state_TN | -3.229 | 0.523 | 184.009 | 37.4 |
| ageCat_under10 | 0.009 | -0.158 | 131.703 | 26.8 |
| state_UT | 0.596 | -0.430 | 127.690 | 26.0 |
| UsCitizen_missing | 2.119 | 2.131 | 125.453 | 25.5 |
| state_PA | 0.997 | 1.152 | 109.007 | 22.2 |
| ssi_missing | 0.025 | 0.354 | 89.686 | 18.3 |
| tanf_missing | 0.509 | 0.499 | 62.073 | 12.6 |
| state_AZ | -0.055 | 0.243 | 54.737 | 11.1 |
| state_IN | 0.395 | 0.341 | 52.161 | 10.6 |
| state_NV | 2.153 | 2.262 | 43.723 | 8.9 |
| ssdi_missing | 0.787 | 0.503 | 41.464 | 8.4 |
| state_MT | -0.638 | -0.484 | 37.203 | 7.6 |
| state_MI | -0.539 | -0.110 | 35.522 | 7.2 |
| state_NM | -2.560 | -2.455 | 34.964 | 7.1 |
| state_DC | -1.445 | -1.004 | 30.348 | 6.2 |
| ssi_no | 1.322 | 1.330 | 24.855 | 5.1 |
| state_AL | -1.121 | -0.681 | 24.838 | 5.1 |
| UsCitizen_yes | -0.403 | -0.407 | 24.774 | 5.0 |
| state_WV | -0.695 | -0.272 | 24.076 | 4.9 |
| houseSize_missing | 0.698 | 0.694 | 23.730 | 4.8 |
| enrollMonth_Jan | -0.147 | -0.144 | 22.046 | 4.5 |
| fedPovLine_missing | -0.369 | -0.348 | 21.670 | 4.4 |
| state_MD | -0.802 | -2.798 | 21.301 | 4.3 |
| tanf_no | 0.037 | 0.033 | 20.458 | 4.2 |
| state_VA | -0.343 | -0.202 | 20.441 | 4.2 |
| ssdi_no | 0.225 | 0.228 | 19.866 | 4.0 |
| state_LA | -0.093 | -0.343 | 19.792 | 4.0 |
| state_DE | 0.498 | -1.414 | 17.956 | 3.7 |
| ageCat_10To17 | 0.097 | -0.080 | 16.775 | 3.4 |
| state_ND | 1.643 | -0.866 | 16.590 | 3.4 |
| ageCat_18To29 | 0.740 | 0.571 | 15.238 | 3.1 |
| speakEnglish_missing | -0.313 | -0.376 | 14.289 | 2.9 |
| state_MS | 0.891 | 1.062 | 13.655 | 2.8 |
| state_ID | 1.130 | 1.266 | 11.345 | 2.3 |
| married_missing | -0.045 | -0.046 | 10.958 | 2.2 |
| speakEnglish_yes | 0.241 | 0.211 | 9.983 | 2.0 |
| state_ME | -1.533 | 0.651 | 9.251 | 1.9 |
| state_HI | -0.817 | -0.372 | 8.894 | 1.8 |
| state_WA | 0.117 | 0.072 | 8.684 | 1.8 |
| cov2016yes_1 | -0.092 | -0.085 | 8.530 | 1.7 |
| state_KS | -0.555 | 1.014 | 8.048 | 1.6 |
| state_VT | -1.308 | 1.721 | 7.905 | 1.6 |
| disabled_no | 0.418 | 0.423 | 7.029 | 1.4 |
| fedPovLine_0To100 | -0.339 | -0.325 | 6.297 | 1.3 |
| race_missing | 0.298 | 0.189 | 5.780 | 1.2 |
| enrollYear_2017 | -0.216 | -0.219 | 5.211 | 1.1 |
| ageCat_30To39 | 0.633 | 0.464 | 5.150 | 1.0 |
| race_hispanic | 0.217 | 0.109 | 4.429 | 0.9 |
| fedPovLine_100To200 | -0.159 | -0.151 | 4.234 | 0.9 |
| married_no | -0.199 | -0.193 | 3.961 | 0.8 |
| houseSize_single | 0.193 | 0.176 | 3.908 | 0.8 |
| ageCat_50To64 | 0.567 | 0.393 | 2.975 | 0.6 |
| race_white | 0.078 | -0.032 | 2.798 | 0.6 |
| houseSize_twoToFive | 0.052 | 0.040 | 2.704 | 0.6 |
| ageCat_40To49 | 0.510 | 0.338 | 2.171 | 0.4 |
| race_asian | 0.095 | -0.063 | 1.810 | 0.4 |
| sex_male | -0.526 | -0.378 | 1.808 | 0.4 |
| race_black | 0.102 | -0.019 | 1.800 | 0.4 |
| sex_female | -0.585 | -0.433 | 1.576 | 0.3 |
| enrollMonth_Dec | 0.013 | 0.024 | 1.569 | 0.3 |
| enrollMonth_Oct | 0.065 | -0.007 | 1.526 | 0.3 |
| enrollMonth_May | -0.091 | -0.006 | 1.515 | 0.3 |
| race_hawaiian | 0.132 | -0.018 | 1.480 | 0.3 |
| enrollMonth_Feb | -0.080 | -0.072 | 1.455 | 0.3 |
| race_native | 0.052 | -0.025 | 1.390 | 0.3 |
| enrollMonth_Sep | 0.004 | 0.002 | 1.344 | 0.3 |
| enrollMonth_Mar | -0.017 | -0.074 | 1.296 | 0.3 |
| enrollMonth_Apr | -0.014 | 0.079 | 1.277 | 0.3 |
| enrollMonth_Jul | -0.021 | 0.021 | 1.257 | 0.3 |
| enrollMonth_Aug | -0.016 | -0.010 | 1.249 | 0.3 |
| enrollMonth_Nov | -0.031 | -0.011 | 1.225 | 0.2 |

**Table 6:** Correlations among top 15 most important predictors for non-emergent acute care visits [top 15 correlations]

- ICD10 code groupers can be found:
  - https://hcup-us.ahrq.gov/toolssoftware/ccsr/dxccsr.jsp
- CPT code groupers can be found:
  - https://data.cms.gov/provider-summary-by-type-of-service/provider-service-classifications/restructured-betos-classification-system
- NDC code groupers can be found:
  - https://www.cms.gov/cciio/programs-and-initiatives/other-insurance-protections/prescription-drug-data-collection
- Clinician specialty code groupers can be found:
  - - https://www.cms.gov/Medicare/Provider-Enrollment-and-Certification/MedicareProviderSupEnroll/downloads/taxonomy.pdf
- Details regarding Area-level SDOH measures can be found in Supplement Methods

| Features | Correlation |
| --- | --- |
| EB009N [Individual therapy] and EB015N [Group therapy] | 0.114 |
| CIR026 [Peripheral and visceral vascular disease] and CIR028 [gangrene] | 0.111 |
| DIG020 [Pancreatic disorders (excluding diabetes)] and DIG017 [Biliary tract disease] | 0.088 |
| DIG019 [Other specified and unspecified liver disease] and DIG017 [Biliary tract disease] | 0.054 |
| DIG019 [Other specified and unspecified liver disease] and CIR031 [Hypotension] | 0.042 |
| Higher rate of substance abuse facilities in county of residence and higher likelihood of losing Medicaid coverage | 0.042 |
| DIG019 [Other specified and unspecified liver disease] and DIG020 [Pancreatic disorders (excluding diabetes)] | 0.036 |
| Number of days with clean air and individual therapy | 0.036 |
| CIR027 [Arterial dissections] and CIR031 [Hypotension] | 0.027 |
| Higher rate of substance abuse facilities in county of residence and EB009N [individual therapy] | 0.025 |
| CIR006 [Pericarditis and pericardial disease] and CIR031 [Hypotension] | 0.024 |
| CIR028 [gangrene] and CIR031 [Hypotension] | 0.024 |
| CIR026 [Peripheral and visceral vascular disease] and CIR031 [Hypotension] | 0.022 |
| E01754430101 [clindamycin (Cleocin)] and EB009N [Individual therapy] | 0.020 |
| CIR031 [Hypotension] and DIG017 [Biliary tract disease] | 0.014 |
| DIG020 [Pancreatic disorders (excluding diabetes)] and CIR031 [Hypotension] | 0.013 |
| CIR026 [Peripheral and visceral vascular disease] and CIR027 [Arterial dissections] | 0.012 |
| CIR027 [Arterial dissections] and CIR006 [Pericarditis and pericardial disease] | 0.012 |
| CIR028 [gangrene] and E01754430101 [clindamycin (Cleocin)] | 0.009 |
| CIR026 [Peripheral and visceral vascular disease] and E01754430101 [clindamycin (Cleocin)] | 0.009 |
| CIR028 [gangrene] and DIG017 [Biliary tract disease] | 0.008 |
| CIR031[Hypotension] and EB009N [Individual therapy] | 0.008 |
| E01754430101[clindamycin (Cleocin)] and goodAirDays | 0.007 |
| CIR025 [Sequela of cerebral infarction and other cerebrovascular disease] and CIR026 [Peripheral and visceral vascular disease] | 0.007 |
| DIG019 [Other specified and unspecified liver disease] and CIR026 [Peripheral and visceral vascular disease] | 0.006 |
| DIG019 [Other specified and unspecified liver disease] and EB009N [Individual therapy] | 0.006 |
| DIG019 [Other specified and unspecified liver disease] and CIR006 [Pericarditis and pericardial disease] | 0.006 |
| DIG019 [Other specified and unspecified liver disease] and stage1_lose_coverage_prob | 0.006 |
| E01754430101 [clindamycin (Cleocin)] and DIG017 [Biliary tract disease] | 0.006 |
| goodAirDays and EB015N [Group therapy] | 0.006 |

**Table 7:** Correlations among top 15 most important predictors for non-emergent acute care visits [top 30 correlations]

- ICD10 code groupers can be found:
  - https://hcup-us.ahrq.gov/toolssoftware/ccsr/dxccsr.jsp
- CPT code groupers can be found:
  - https://data.cms.gov/provider-summary-by-type-of-service/provider-service-classifications/restructured-betos-classification-system
- NDC code groupers can be found:
  - https://www.cms.gov/cciio/programs-and-initiatives/other-insurance-protections/prescription-drug-data-collection
- Clinician specialty code groupers can be found:
  - - https://www.cms.gov/Medicare/Provider-Enrollment-and-Certification/MedicareProviderSupEnroll/downloads/taxonomy.pdf
- Details regarding Area-level SDOH measures can be found in Supplement Methods

| Features | Correlation |
| --- | --- |
| CIR033 [Acute phlebitis; thrombophlebitis and thromboembolism] and CIR034 [Chronic phlebitis; thrombophlebitis and thromboembolism] | 0.121 |
| EB015N [Group therapy] and EB009N [Individual therapy] | 0.114 |
| CIR026 [Peripheral and visceral vascular disease] and CIR028 [Gangrene] | 0.111 |
| DIG019 [Other specified and unspecified liver disease] and DIG016 [Peritonitis and intra-abdominal abscess] | 0.065 |
| CIR026 [Peripheral and visceral vascular disease] and CIR032 [Other specified and unspecified circulatory disease] | 0.063 |
| DIG020 [Pancreatic disorders (excluding diabetes)] and DIG016 [Peritonitis and intra-abdominal abscess] | 0.050 |
| DIG019 [Other specified and unspecified liver disease] and CIR031 [Hypotension] | 0.042 |
| EB015N [Group therapy] and RT020N [Physical, occupational and speech therapy] | 0.037 |
| CIR028 [Gangrene] and CIR032 [Other specified and unspecified circulatory disease] | 0.037 |
| DIG019 [Other specified and unspecified liver disease] and DIG020 [Pancreatic disorders (excluding diabetes)] | 0.036 |
| DIG019 [Other specified and unspecified liver disease] and PO000O [Other organ system procedure] | 0.030 |
| DIG016 [Peritonitis and intra-abdominal abscess] and CIR031 [Hypotension] | 0.028 |
| CIR027 [Arterial dissections] and CIR031 [Hypotension] | 0.027 |
| DIG016 [Peritonitis and intra-abdominal abscess] and CIR033 [Acute phlebitis; thrombophlebitis and thromboembolism] | 0.025 |
| DC002N [DME – oxygen and supplies] and CIR031 [Hypotension] | 0.025 |
| PO000O [Other organ system procedure] and EB009N [Individual therapy] | 0.024 |
| CIR028 [Gangrene] and CIR031 [Hypotension] | 0.024 |
| DC002N [DME – oxygen and supplies] and PO000O [Other organ system procedure] | 0.022 |
| CIR026 [Peripheral and visceral vascular disease] and CIR031 [Hypotension] | 0.022 |
| DIG019 [Other specified and unspecified liver disease] and CIR033 [Acute phlebitis; thrombophlebitis and thromboembolism] | 0.021 |
| CIR033 [Acute phlebitis; thrombophlebitis and thromboembolism] and CIR031 [Hypotension] | 0.021 |
| CIR026 [Peripheral and visceral vascular disease] and CIR033 [Acute phlebitis; thrombophlebitis and thromboembolism] | 0.019 |
| DC002N [DME – oxygen and supplies] and CIR033 [Acute phlebitis; thrombophlebitis and thromboembolism] | 0.016 |
| DIG020 [Pancreatic disorders (excluding diabetes)] and CIR033 [Acute phlebitis; thrombophlebitis and thromboembolism] | 0.016 |
| CIR033 [Acute phlebitis; thrombophlebitis and thromboembolism] and PO000O [Other organ system procedure] | 0.016 |
| RT020N [Treatment – physical, occupational, speech therapy] and EB009N [Individual therapy] | 0.015 |
| CIR031 [Hypotension] and PO000O [Other organ system procedure] | 0.015 |
| CIR026 [Peripheral and visceral vascular disease] and DC002N [DME – oxygen and supplies] | 0.015 |
| DIG019 [Other specified and unspecified liver disease] and DC002N [DME – oxygen and supplies] | 0.014 |
| DIG020 [Pancreatic disorders (excluding diabetes)] and CIR031 [Hypotension] | 0.013 |

**Table 8:** Comparative effectiveness of models predicting patient cost*^


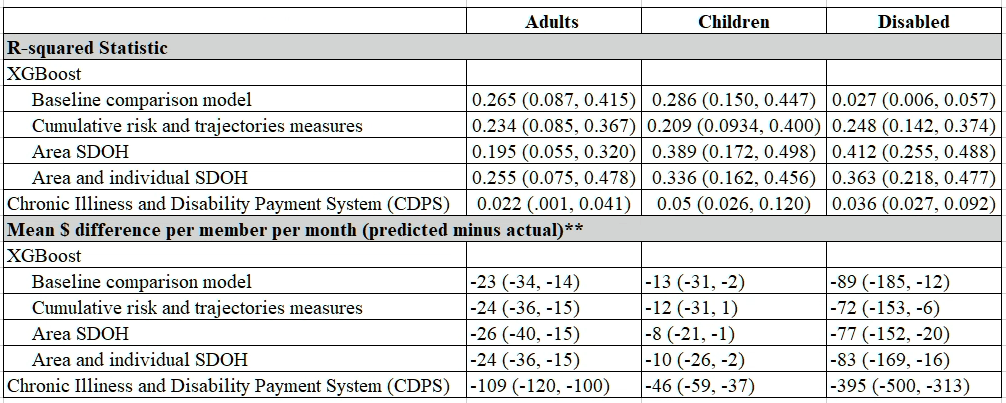


*Based on the R-squared statistic, for adults, the best performing model was the baseline comparison model; for children, the area and individual SDOH measures model; for individuals with disabilities, the area SDOH measures only model.

^95% CI created via bootstrapping procedure described in manuscript

**A negative value indicates the predicted $ per member per month was lower than the actual $ per member per month

**Table 9:** Assessment of racial bias for predicting patient cost^*


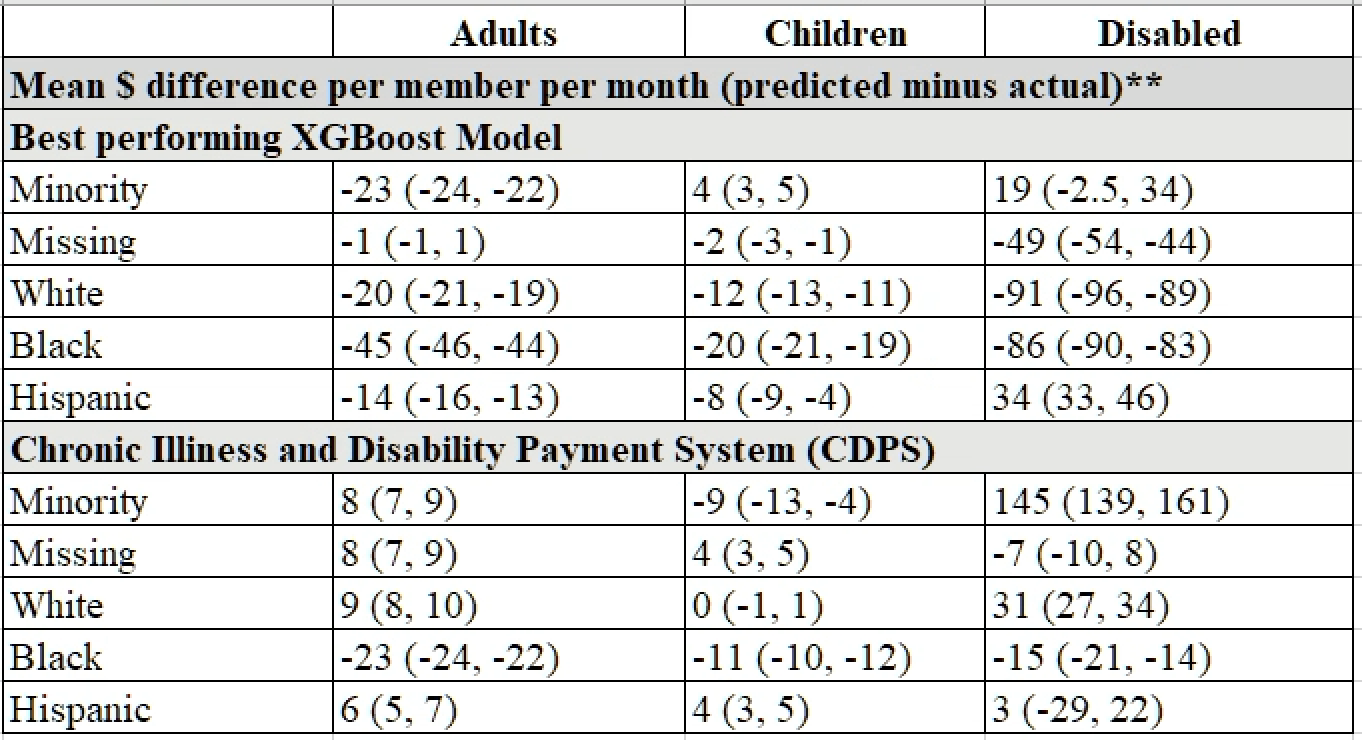


^Cost measures is the mean difference in $ per member per month (predicted minus actual). 95% CI constructed via bootstrapping procedure described in manuscript.

*We selected the best performing model for each subgroup based on findings in Supplement Table 7. For adults, it was the baseline comparison model; for children, it was the area and individual SDOH model; for individuals with disabilities, it was the area SDOH model.

**A negative value indicates the predicted $ per member per month was lower than the actual $ per member per month

**Table 10:** Assessment of racial bias. Point estimates are shown for each outcome metric, with 95% confidence intervals in parentheses.

|  | **Non-emergent acute care visits** | | | **All-cause acute care visits** | | |
| --- | --- | --- | --- | --- | --- | --- |
| **Race** | **Sensitivity** | **Specificity** | **F1 Score** | **Sensitivity** | **Specificity** | **F1 Score** |
| Other | 0.063 (0.059, 0.066) | 0.999 (0.998, 1.000) | 0.117 (0.110, 0.118) | 0.245 (0.244, 0.250) | 0.991 (0.990, 0.992) | 0.380 (0.375, 0.384) |
| Missing | 0.104 (0.013, 0.016) | 0.997 (0.996, 0.998) | 0.185 (0.183, 0.186) | 0.305 (0.303, 0.307) | 0.982 (0.981, 0.983) | 0.444 (0.442, 0.444) |
| White | 0.089 (0.088, 0.091) | 0.998 (0.997, 0.999) | 0.161 (0.161, 0.163) | 0.284 (0.283, 0.287) | 0.984 (0.983, 0.985) | 0.423 (0.422, 0.424) |
| Black | 0.097 (0.096, 0.099) | 0.997 (0.996, 0.998) | 0.174 (0.173, 0.176) | 0.317 (0.316, 0.319) | 0.976 (0.975, 0.976) | 0.457 (0.456, 0.458) |
| Hispanic | 0.065 (0.064, 0.068) | 0.998 (0.997, 0.999) | 0.121 (0.118, 0.122) | 0.242 (0.240, 0.245) | 0.989 (0.988, 0.990) | 0.375 (0.372, 0.376) |

**Table 11:** Sensitivity analysis for assessment of racial bias when race/ethnicity was eliminated as a predictor from the model. Point estimates are shown for each outcome metric, with 95% confidence intervals in parentheses, for the best performing model by MCC.

1. Overall population

|  | **All-cause** | **Non-emergent** |
| --- | --- | --- |
| AUC | 0.793 (0.791, 0.804) | 0.791 (0.780, 0.794) |
| Accuracy | 0.836 (0.834, 0.840) | 0.902 (0.899, 0.904) |
| MCC | 0.419 (0.410, 0.429) | 0.255 (0.232, 0.262) |
| Sensitivity | 0.289 (0.280, 0.297) | 0.092 (0.082, 0.097) |
| Specificity | 0.982 (0.981, 0.984) | 0.998 (0.997, 0.999) |
| NPV | 0.838 (0.836, 0.842) | 0.903 (0.900, 0.905) |
| PPV | 0.809 (0.800, 0.825) | 0.823 (0.774, 0.835) |
| F1 Score | 0.426 (0.416, 0.437) | 0.160 (0.148, 0.173) |

1. By race/ethnicity

|  | **Non-emergent acute care visits** | | | **All-cause acute care visits** | | |
| --- | --- | --- | --- | --- | --- | --- |
| **Race** | **Sensitivity** | **Specificity** | **F1 Score** | **Sensitivity** | **Specificity** | **F1 Score** |
| Other | 0.069 (0.065, 0.071) | 0.998 (0.998, 0.999) | 0.127 (0.125, 0.132) | 0.245 (0.240, 0.247) | 0.990 (0.989, 0.991) | 0.378 (0.376, 0.382) |
| Missing | 0.105 (0.103, 0.106) | 0.997 (0.996, 0.998) | 0.186 (0.183, 0.186) | 0.306 (0.304, 0.307) | 0.982 (0.981, 0.983) | 0.446 (0.445, 0.447) |
| White | 0.087 (0.086, 0.089) | 0.997 (0.996, 0.998) | 0.157 (0.156, 0.159) | 0.287 (0.286, 0.288) | 0.982 (0.982, 0.983) | 0.423 (0.422, 0.423) |
| Black | 0.088 (0.086, 0.089) | 0.997 (0.996, 0.998) | 0.158 (0.157, 0.161) | 0.301 (0.299, 0.303) | 0.977 (0.976, 0.978) | 0.440 (0.438, 0.441) |
| Hispanic | 0.064 (0.061, 0.065) | 0.998 (0.997, 0.999) | 0.119 (0.116, 0.120) | 0.242 (0.239, 0.245) | 0.987 (0.986, 0.988) | 0.374 (0.373, 0.377) |

**Table 12:** Sensitivity analysis for assessment of racial bias after downsampling White patients. Point estimates are shown for each outcome metric, with 95% confidence intervals in parentheses, for the best performing model by MCC*

*White patients consisted of 40% of all patients in our original sample while Black patients consisted of 17% of all patients. We explored reducing White patients to roughly 20% and 30% of the total sample. To accelerate computation, we used a random sample of 1 million patients.

Non-emergent Acute Care Visits

|  | **White patients consisting of 20% of total sample** | | | **White patients consisting of 30% of total sample** | | |
| --- | --- | --- | --- | --- | --- | --- |
| **Race** | **Sensitivity** | **Specificity** | **F1 Score** | **Sensitivity** | **Specificity** | **F1 Score** |
| Other | 0.038 (0.031, 0.039) | 0.995 (0.994, 0.996) | 0.070 (0.062, 0.076) | 0.056 (0.051, 0.059) | 0.999 (0.998, 1.000) | 0.105 (0.086, 0.107) |
| Missing | 0.083 (0.081, 0.085) | 0.994 (0.993, 0.995) | 0.147 (0.143, 0.150) | 0.075 (0.072, 0.076) | 0.995 (0.994, 0.996) | 0.134 (0.130, 0.139) |
| White | 0.063 (0.061, 0.064) | 0.995 (0.993, 0.995) | 0.113 (0.110, 0.115) | 0.061 (0.060, 0.064) | 0.995 (0.994, 0.996) | 0.110 (0.107, 0.115) |
| Black | 0.081 (0.078, 0.082) | 0.993 (0.992, 0.994) | 0.144 (0.140, 0.147) | 0.060 (0.058, 0.062) | 0.992 (0.991, 0.993) | 0.108 (0.100, 0.109) |
| Hispanic | 0.047 (0.043, 0.048) | 0.995 (0.994, 0.996) | 0.085 (0.079, 0.087) | 0.032 (0.030, 0.034) | 0.996 (0.995, 0.997) | 0.060 (0.054, 0.062) |

**Table 13:** Sensitivity analysis for prediction of acute care use among adults, when children (people <18 years of age at time of first eligibility) are removed from the model dataset. Point estimates are shown for each outcome metric, with 95% confidence intervals in parentheses, for the best performing model by MCC.

|  | **All-cause** | | **Non-emergent** | |
| --- | --- | --- | --- | --- |
|  | Adults only | Everyone  (under 18 included) | Adults only | Everyone  (under 18 included) |
| AUC | 0.829 (0.825, 0.833) | 0.807 (0.800, 0.809) | 0.807 (0.797, 0.811) | 0.793 (0.790, 0.795) |
| Accuracy | 0.836 (0.833, 0.840) | 0.838 (0.835, 0.841) | 0.906 (0.904, 0.909) | 0.903 (0.899, 0.904) |
| MCC | 0.469 (0.460, 0.479) | 0.419 (0.418, 0.437) | 0.278 (0.264, 0.292) | 0.287 (0.273, 0.300) |
| Sensitivity | 0.359 (0.349, 0.368) | 0.287 (0.285, 0.302) | 0.110 (0.102, 0.118) | 0.107 (0.103, 0.116) |
| Specificity | 0.977 (0.975, 0.978) | 0.982 (0.981, 0.984) | 0.997 (0.997, 0.998) | 0.998 (0.997, 0.999) |
| NPV | 0.838 (0.835, 0.842) | 0.841 (0.836, 0.843) | 0.908 (0.905, 0.910) | 0.903 (0.900, 0.905) |
| PPV | 0.819 (0.808, 0.829) | 0.810 (0.807, 0.834) | 0.832 (0.787, 0.843) | 0.810 (0.780, 0.841) |
| F1 Score | 0.194 (0.181, 0.208) | 0.432 (0.422, 0.442) | 0.426 (0.415, 0.436) | 0.155 (0.143, 0.168) |

**Table 14:** Sensitivity analysis for Random Forest using a downsampling procedure for reducing the number of patients with no acute care use. Point estimates are shown for each outcome metric, with 95% confidence intervals in parentheses, for the best performing model by MCC*

*Best performing Random Forest Model was the ‘Cumulative Risk and Risk Trajectories’ model, as indicated by MCC. We note that downsampling was less necessary for all-cause acute care visits compared to non-emergent visits, as patients with at least 1 all-cause acute care visit represented 20 percent of our sample compared to 10 percent of patients with at least 1 non-emergent acute care visit.

**Non-emergent:**

|  | **Main Analysis Sample**  (roughly 10% of patients had at least 1 non-emergent acute care visit in sample) | **Sample 1**  (roughly 25% of patients had at least 1 non-emergent acute care visit in sample) | **Sample 2**  (roughly 50% of patients had at least 1 non-emergent acute care visit in sample) |
| --- | --- | --- | --- |
| AUC | 0.750 (0.740, 0.754) | 0.762 (0.759, 0.764) | 0.763 (0.760, 0.765) |
| Accuracy | 0.897 (0.894, 0.899) | 0.888 (0.887, 0.889) | 0.634 (0.632, 0.635) |
| MCC | 0.169 (0.140, 0.170) | 0.291 (0.285, 0.296) | 0.233 (0.229, 0.235) |
| Sensitivity | 0.035 (0.027, 0.037) | 0.269 (0.264, 0.272) | 0.754 (0.749, 0.758) |
| Specificity | 0.999 (0.998, 1.000) | 0.961 (0.960, 0.962) | 0.620 (0.618, 0.621) |
| NPV | 0.897 (0.894, 0.900) | 0.917 (0.916, 0.918) | 0.955 (0.954, 0.956) |
| PPV | 0.920 (0.830, 0.924) | 0.451 (0.445, 0.459) | 0.190 (0.189, 0.192) |
| F1 Score | 0.061 (0.058, 0.076) | 0.337 (0.323, 0.349) | 0.303 (0.296, 0.311) |

**All-cause:**

|  | **Main Analysis Sample**  (roughly 20% of patients in sample had at least 1 non-emergent acute care visit) | **Sample 1**  (35% of patients in sample had at least 1 non-emergent acute care visit) | **Sample 2**  (50% of patients in sample had at least 1 non-emergent acute care visit) |
| --- | --- | --- | --- |
| AUC | 0.746 (0.745, 0.758) | 0.759 (0.758, 0.762) | 0.759 (0.757, 0.761) |
| Accuracy | 0.817 (0.815, 0.822) | 0.808 (0.807, 0.810) | 0.726 (0.724, 0.727) |
| MCC | 0.313 (0.312, 0.333) | 0.357 (0.356, 0.364) | 0.330 (0.327, 0.333) |
| Sensitivity | 0.164 (0.160, 0.174) | 0.391 (0.390, 0.396) | 0.627 (0.625, 0.632) |
| Specificity | 0.990 (0.989, 0.993) | 0.919 (0.918, 0.921) | 0.752 (0.750, 0.753) |
| NPV | 0.817 (0.814, 0.820) | 0.850 (0.849, 0.852) | 0.884 (0.883, 0.886) |
| PPV | 0.820 (0.819, 0.858) | 0.562 (0.561, 0.570) | 0.402 (0.399, 0.406) |
| F1 Score | 0.273 (0.269, 0.289) | 0.461 (0.454, 0.473) | 0.490 (0.483, 0.498) |

**Figure 1:** Comparative effectiveness of models predicting utilization of non-emergent acute care visits*^


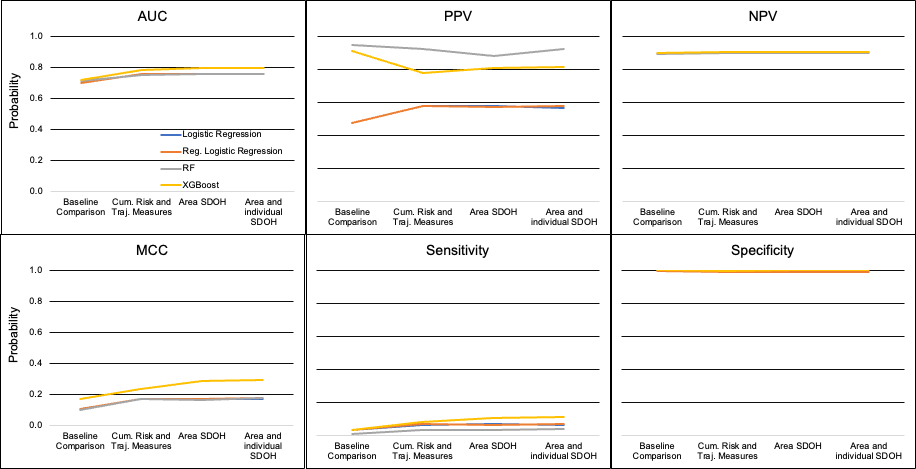


Abbreviations: Area under the ROC Curve (AUC); Matthews correlation coefficient (MCC); Negative predictive value (NPV); Positive predictive value (PPV); Extreme gradient boosting (XGBoost); Random Forest (RF).

*We sequentially compared multiple alternative models to compare the benefits of including different predictor variables and the impact of alternative model fitting algorithms. First, we devised a model with demographics and clinical history to mimic standard risk models (named ‘baseline comparison model’). Second, we developed a model with cumulative risk and risk trajectories variables (named the ‘cumulative risk and trajectories’ model). Finally, we created two additional models (including all measures in the ‘cumulative risk and trajectories’ model), one with area-level SDOH predictors (named, ‘area SDOH’) and another with both area- and individual-level SDOH predictors (named ‘area and individual SDOH’). See **Supplement** for model specification. See **Supplement Table 6** for point estimates and 95% confidence intervals for each performance measure.

**^^^**Predictors were measured in the six-month period after a patient’s first month of enrollment in Medicaid from 2017-18. The outcome was a binary (yes/no) indicator of whether a patient had at least 1 non-emergent acute care visit in the 6-month period immediately following the 6-month predictor measurement period. See **Supplement** for machine learning modeling.

**Figure 2:** Comparative effectiveness of models predicting utilization of all-cause acute care visits*^


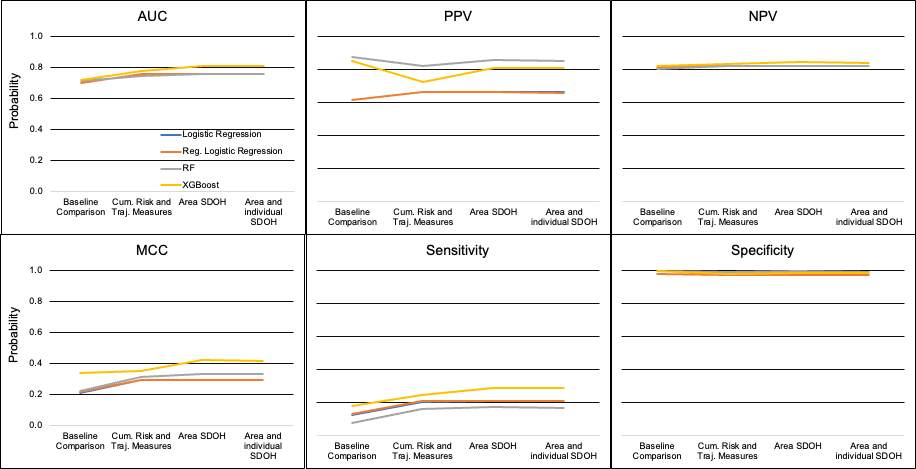


Abbreviations: Area under the ROC Curve (AUC); Matthews correlation coefficient (MCC); Negative predictive value (NPV); Positive predictive value (PPV); Extreme gradient boosting (XGBoost); Random Forest (RF)

*We sequentially compared multiple alternative models to compare the benefits of including different predictor variables and the impact of alternative model fitting algorithms. First, we devised a model with demographics and clinical history to mimic standard risk models (named ‘baseline comparison model’). Second, we developed a model with cumulative risk and risk trajectories variables (named the ‘cumulative risk and trajectories’ model). Finally, we created two additional models (including all measures in the ‘cumulative risk and trajectories’ model), one with area-level SDOH predictors (named, ‘area SDOH’) and another with both area- and individual-level SDOH predictors (named ‘area and individual SDOH’). See **Supplement** for model specification. See **Supplement Table 7** for point estimates and 95% confidence intervals for each performance measure.

**^^^**Predictors were measured in the six-month period after a patient’s first month of enrollment in Medicaid from 2017-18. The outcome was a binary (yes/no) indicator of whether a patient had at least 1 all-cause acute care visit in the 6-month period immediately following the 6-month predictor measurement period. See **Supplement** for machine learning modeling.

**Figure 3:** Importance of top 15 predictors in the best performing model (XGBoost model with clinical, cumulative risk and trajectories, and area-level SDOH measures)*^


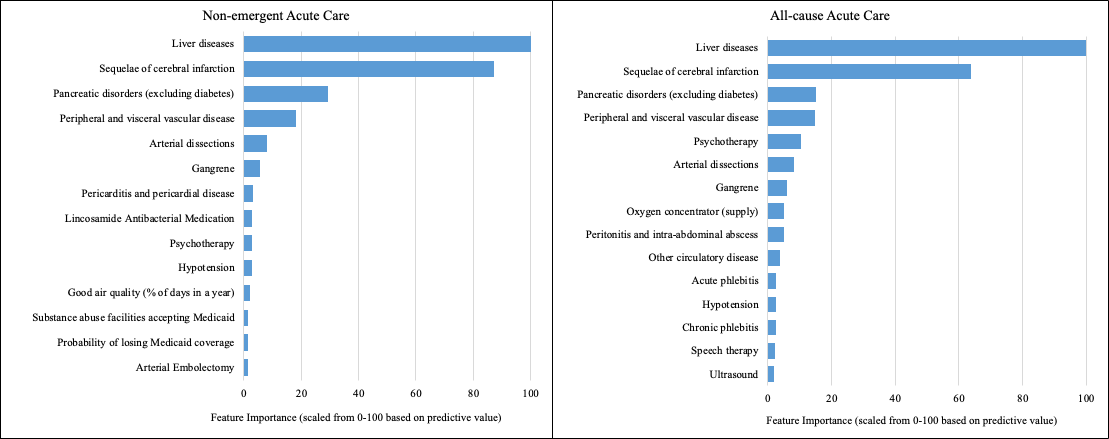


*XGBoost model with clinical, cumulative risk and trajectories, and area-level SDOH measures was the best performing model for predicting both all-cause and non-emergent acute care utilization.

^Feature importance was computed using the “gain” importance type function (Gini index). The feature importance value was scaled from 0-100 based on predictive value.
